# A switchable self-assembling and disassembling chiral system based on a porphyrin-substituted phenylalanine–phenylalanine motif

**DOI:** 10.1038/ncomms12657

**Published:** 2016-09-01

**Authors:** Georgios Charalambidis, Evangelos Georgilis, Manas K. Panda, Christopher E. Anson, Annie K. Powell, Stephen Doyle, David Moss, Tobias Jochum, Peter N. Horton, Simon J. Coles, Mathieu Linares, David Beljonne, Jean-Valère Naubron, Jonas Conradt, Heinz Kalt, Anna Mitraki, Athanassios G. Coutsolelos, Teodor Silviu Balaban

**Affiliations:** 1 Department of Chemistry, Bioinorganic Chemistry Laboratory, University of Crete, Vassilika Vouton, Heraklion, 70013 Crete, Greece; 2 Department of Materials Science and Technology, University of Crete, Vassilika Vouton, Heraklion, 70013 Crete, Greece; 3 Institute of Electronic Structure and Laser (I.E.S.L.) Foundation for Research and Technology Hellas (FO.R.T.H.) Vassilika Vouton, Heraklion, 70013 Crete, Greece; 4 Karlsruhe Institute of Technology (KIT), Institute of Inorganic Chemistry, Engesserstrasse 15, D-76131 Karlsruhe, Germany; 5 Karlsruhe Institute of Technology (KIT), Institute for Nanotechnology (INT), Hermann-von-Helmholtz-Platz 1, D-76344 Eggenstein-Leopoldshafen, Germany; 6 Karlsruhe Institute of Technology, Institute for Synchrotron Radiation and ANKA, Hermann-von-Helmholtz-Platz 1, D-76344 Eggenstein-Leopoldshafen, Germany; 7 School of Chemistry, EPSRC National Crystallography Service, University of Southampton, Highfield, Southampton SO17 1BJ, UK; 8 Department of Physics, Chemistry and Biology, Linköping University, SE-581 83 Linköping, Sweden; 9 Swedish e-Science Research Centre (SeRC), Linköping University, SE-581 83 Linköping, Sweden; 10 Département de Chimie, Chimie des Matériaux Nouveaux and Centre d'Innovation et de Recherche en Matériaux Polymères, Université de Mons—UMONS/Materia Nova, Place du Parc, 20, B-7000 Mons, Belgium; 11 Aix Marseille Université, CNRS FR 1739, Spectropole, Avenue Escadrille Normandie Niemen, F-13397 Marseille, France; 12 Karlsruhe Institute of Technology (KIT), Institute of Applied Physics and Center for Functional Nanostructures (CFN), D-76131 Karlsruhe, Germany; 13 Aix Marseille Univ, CNRS, Centrale Marseille, Institut des Sciences Moléculaires de Marseille (iSm2), UMR 7313, Chirosciences, Avenue Escadrille Normandie Niemen, Service 442, F-13397 Marseille, France

## Abstract

Artificial light-harvesting systems have until now not been able to self-assemble into structures with a large photon capture cross-section that upon a stimulus reversibly can switch into an inactive state. Here we describe a simple and robust **F**_*L*_**F**_*L*_-dipeptide construct to which a *meso*-tetraphenylporphyrin has been appended and which self-assembles to fibrils, platelets or nanospheres depending on the solvent composition. The fibrils, functioning as quenched antennas, give intense excitonic couplets in the electronic circular dichroism spectra which are mirror imaged if the unnatural **F**_*D*_**F**_*D*_-analogue is used. By slightly increasing the solvent polarity, these light-harvesting fibres disassemble to spherical structures with silent electronic circular dichroism spectra but which fluoresce. Upon further dilution with the nonpolar solvent, the intense Cotton effects are recovered, thus proving a reversible switching. A single crystal X-ray structure shows a head-to-head arrangement of porphyrins that explains both their excitonic coupling and quenched fluorescence.

## Abstract (web summary)

Artificial light-harvesting systems cannot self-assemble into photon-capturing structures that can reversibly switch into an inactive state. Here, the authors describe a simple and robust dipeptide construct which self-assembles to fibrils, platelets or nanospheres with varying optical properties.

## Graphical abstract

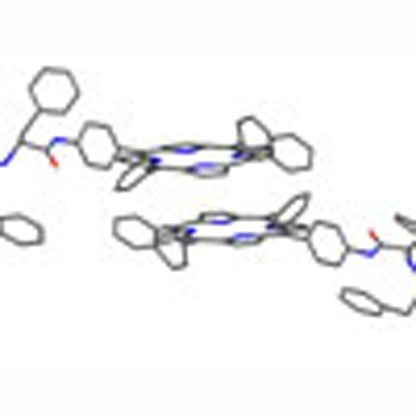

Artificial self-assembling light-harvesting systems are of interest in dye-sensitized solar cells where a large photon capture cross section can assure that efficient energy transfer occurs to a large band-gap semiconductor^1^. Such cells should be able to function also under dim light, such as at dusk or dawn, and could be of use for indoor applications or even outside at night in cities where radiant billboards are abundant. The chlorosomes are the antenna system of various green, brown or some filamentous bacteria that live in deep waters being able to scavenge photons and thus gain energy photosynthetically where other forms of life must rely on different metabolisms^2^,^3^,^4^. One of the present authors was involved in mimicking the chlorosomal antenna with fully synthetic pigments^5^, whereas other groups have used semisynthetic approaches, mainly starting from algal chlorophyll *a* (Chl *a*), to endow chromophores with self-assembling ability^5^. Self-assembling chromophores are currently an active research area with diverse applications, among which are tubular antenna systems of cyanine dyes^6^,^7^,^8^.

Early in evolution, after bacteriochlorophyll (BChl) biosynthetic pathways were optimized, a considerable economy of genes expressing large proteins could be realized by using self-assembly for constructing light-harvesting apparata which are adapted to the illumination conditions of the habitat. Self-assembling antennas can be constructed at three levels of complexity. First, at the simplest level, chlorosomes in green photosynthetic bacteria constitute a very large antenna formed by self-assembled BChl *c*, *d* or *e*, without using polypeptides or proteins to organize the chromophores in precise orientations^2^,^3^,^4^. At the second and more sophisticated level, functional circular arrangements are encountered in the antenna systems LH1 and LH2 of purple photosynthetic bacteria^9^,^10^,^11^. These are formed by self-assembly within the photosynthetic membrane of small polypetides having ∼54 amino acids that bind the Mg atoms of BChl *a.* At the third, and most complex level, different protein subunits self-assemble into tightly bound protein complexes. Higher plants use the two photosystems (PS) I and II, which are multiproteinic complexes comprising 96 and 36 chlorophyllous pigments, respectively, as well as carotenoids^12^. In addition, multiple copies of their associated light-harvesting complexes (LHC I, LHC II) assure a vectorial energy transfer from an initially excited antenna chlorophyll to the special pairs P700 (for PS I) or P680 (for PS II) within the reaction centres.

While there is great diversity in photosynthetic antenna systems, the reaction centres have strikingly similar architectures and operating principles for stabilizing the electron and the hole on opposite sides of the membrane. However, under strong illumination conditions, excess radiation can rapidly damage the photosystems, in response to which higher plants have devised the ingenious mechanism of non-radiative photochemical quenching^13^,^14^.

To devise more robust artificial systems, the blueprint offered by nature should be well understood, but does not need to be identically copied^15^,^16^,^17^,^18^. For instance, although synthesizing polypeptides with ∼50 amino acids is feasible, such constructs will never be applicable in large-scale devices. Thus in a viable approach, the function of reaction centres can be provided by wide band gap semiconductors and their antennas could be assembled from a very short peptide binding strongly to absorbing chromophores. Currently, all artificial antenna systems, be they assembled via covalent syntheses, as in elegant dendrimeric architectures^19^, or self-assembling BChl *c* mimics^5^, once formed cannot be dismantled. In this article, we present a simple porphyrin decorating a self-assembling chiral system that can reversibly change its morphology via slight changes in the solvent composition from a passive state with intense excitonic couplets in the electronic circular dichroism spectra (ECD) and quenched fluorescence, to an active state with intense fluorescence but with silent ECD spectra.

Aromatic dipeptides have high self-assembling propensities. The self-assembly of both isomers of diphenylalanine (**F**_*L*_**F**_*L*_ and **F**_*D*_**F**_*D*_) into tubular or spherical forms was first reported in 2003 by Reches and Gazit^20^, and Song *et al.*^21^. Moreover, in a very recent study, a systematic approach using coarse-grained molecular dynamics calculations identified the highest aggregation propensities for the **FF**, **FW** and **WF** (where **W** stands for tryptophan) motifs in dipeptides and tripeptides^22^. The most experimentally scrutinized to date remain the **F**_*L*_**F**_*L*_-based assemblies^23^,^24^,^25^,^26^,^27^,^28^,^29^,^30^,^31^,^32^ and have shown promising luminescence^33^, sensing^25^ or medical applications^34^. N-terminal protecting groups such as Fmoc play an important role in diphenylalanine assemblies. The **Fmoc-F**_*L*_**F**_*L*_ dipeptides self-assemble into fibrous hydrogels and the self-assembly was proposed to be driven by interlocked antiparallel sheets and *π-*stacking interactions between the fluorenyl groups of Fmoc^29^. Subsequently, the role of the Fmoc moiety in self-assembly was further investigated^35^.

**F**_*L*_**F**_*L*_ assemblies have proven to have extreme mechanical properties rivalling silk, spider's web, Kevlar or other aramid fibres, being among the toughest of all-organic materials^20^,^31^. Non-covalent incorporation of a water-soluble porphyrin into **FF-**nanotubes was recently described as a biomimetic antenna system^33^ as well as being active for photocatalytic water oxidation^36^. We have gone a step further in complexity having previously prepared the **FF-**porphyrin conjugates by covalently linking *meso*-tetraphenylporphyrin (**TPP**) to the C-terminus of **F**_*L*_**F**_*L*_ (ref. 37).

In the present work, we report the formation of fibrous assemblies formed by these **FF-TPP** conjugates on dilution from dichloromethane into heptane. We structurally characterize these assemblies using SEM, FTIR, powder diffraction as well as ultraviolet-Vis and ECD spectroscopy, and stationary and time-resolved fluorescence. For the **Fmoc-F**_*L*_**F**_*L*_**-TPP** conjugate, we are able to obtain single microcrystals and solve their structure, revealing a J-aggregate arrangement. **FF** is a versatile motif which we demonstrate can assemble reversibly with porphyrins to give large antennas. Furthermore, we synthesize the unnatural **X-F**_*D*_**F**_*D*_**-TPP** conjugates (where X is H, Fmoc or Boc) and study the transfer of chirality from the molecular to the supramolecular level, which is currently a topic of interest^38^,^39^.

## Results

### Nanomorphology

**Figure 1:**
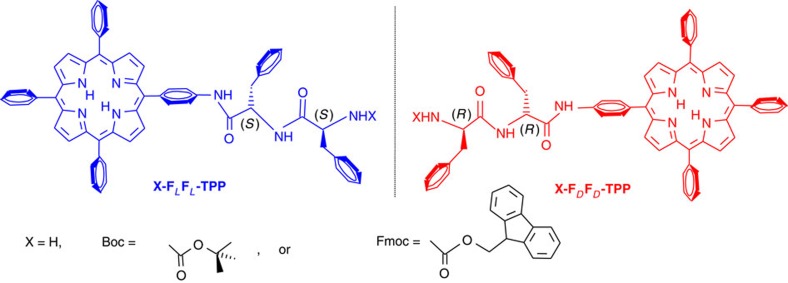
Formulae of compounds addressed in this work. **F**_*L*_**F**_*L*_ is the natural dipeptide while **F**_*D*_**F**_*D*_ is its mirror image. In the unprotected version X=H, while Boc stands for *tert*-butoxycarbonyl and Fmoc stands for 9-fluorenylmethoxycarbonyl.

Nanosphere formation of the **F**_*L*_**F**_*L*_**-TPP** or **F**_*D*_**F**_*D*_**-TPP** conjugates occurs readily on dissolving these in 1,1,1,3,3,3-hexafluoro-2-propanol (HFIP) and then diluting with a polar solvent such as water, tetrahydrofuran, methanol, ethanol or acetonitrile with a HFIP: polar solvent ratio of 2:8 (ref. 37). In this previous study we have additionally synthesized the conjugates **Fmoc-F**_*L*_**F**_*L*_**-TPP** and **Boc- F**_*L*_**F**_*L*_**-TPP** and showed that they self-assemble into spheres in the aforementioned solvents. The optimized reactions have high yields, the diphenylalanine dipeptides can be obtained commercially and the starting meso-(4-aminophenyl)-triphenylporphyrin (TPP-NH_2_) can be obtained either commercially or synthesized on the 10 g scale in a standard laboratory. Figure 1 presents the constructs addressed in this work.

By modifying the solvent conditions, and dissolving the porphyrin conjugates in anhydrous dichloromethane, which is a ‘good solvent', then ‘injected' into a dry nonpolar solvent such as *n*-heptane, *n*-hexane or cyclohexane (consequently termed ‘bad solvent') we now observe broadened, red-shifted and split Soret bands typical of porphyrinic *J*-aggregates^40^, which are named after Jelley^41^. We find such chromophores to be assembled in staircase-like architectures, usually exhibiting sharp, red-shifted absorption maxima.

**Figure 2:**
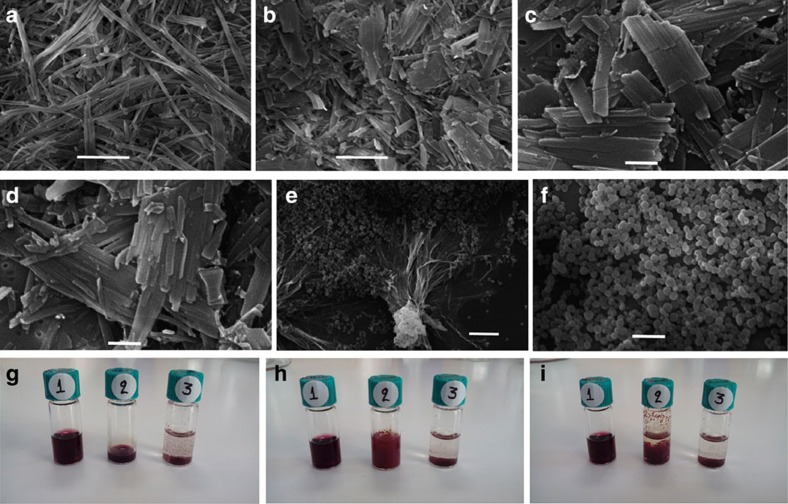
Changes in the morphology of Fmoc-F_*L*_F_*L*_-TPP with the solvent composition. Samples observed in SEM following critical point drying of **Fmoc-F**_*L*_**F**_*L*_**-TPP** dissolved in dry dichloromethane which was injected into *n-*heptane at room temperature. Final relative ratios (v/v) of CH_2_Cl_2_ to *n-*heptane were: (**a**) 3:7; (**b**) 2:8; (**c**) and (**d**) same sample as in **b** imaged at higher magnification with sideways associated fibrils. (**e**,**f**) 5:5. Further SEM micrographs at 3:7 and 2:8 concentrations are shown in the Supplementary Fig. 19. Scale bars, 5 μm in **a**,**b**,**e**; 2 μm in **f**; 1 μm in **c**,**d**. Pictures of vials showing **Fmoc-F**_*L*_**F**_*L*_**-TPP** in the following conditions: (**g**) immediately after addition of: 1 ml pure dichloromethane (vial 1); 0.2 ml pure dichloromethane (vial 2); 1 ml pure *n-*heptane (vial 3). (**h**) immediately after addition of 0.8 ml heptane to vial 2. (**i**) Twenty-four hours after addition of 0.8 ml *n*-heptane to vial 2. The final concentration of **Fmoc-F**_*L*_**F**_*L*_**-TPP** in all conditions was 1 mM. A clear solution is observed for the sample dissolved in pure dichloromethane, while in pure *n*-heptane a rapidly sedimenting precipitate is formed. Following addition of *n-*heptane to the dichloromethane-dissolved sample in vial 2, turbidity rapidly ensues and a slowly sedimenting precipitate is formed.

Instead of nanospheres, we recorded the formation of micrometre long fibrils in the case when the dichloromethane: *n*-heptane ratio was 3:7, as shown in Fig. 2a. With a ratio of 2:8, larger platelets appear which are not hollow, as inferred from the SEM images, and seem to consist of sideways-associated fibrils (Fig. 2b–d). Due to the fact that the fibrillary structures appear in bundles it is very difficult to distinguish and measure the dimensions of isolated fibrils; nevertheless most of them present a width of about 200 nm and they can reach several micrometres in length.

With a 1:1 ratio of dichloromethane to *n*-heptane, we observed spheres with diameter ranging from 400 to 600 nm coexisting with fibrils (Fig. 2e,f). The picture in Fig. 2g shows vials containing **Fmoc-F**_*L*_**F**_*L*_**-TPP** at 1 mM final concentration immediately after addition of either 1 ml pure dichloromethane or 1 ml pure *n-*heptane. A clear solution is observed for the sample dissolved in pure dichloromethane, while in pure *n-*heptane, the sample is completely insoluble and rapidly sediments at the bottom. We further examined both samples with SEM. A drop of the clear bordeaux-magenta coloured dichloromethane solution forms an amorphous film on deposition and evaporation on the glass slide, shown also in SEM (Supplementary Fig. 19). The sediments in the *n-*heptane solution are imaged as quasi-spherical agglomerates from which small platelet-like morphologies seem to protrude. The dichloromethane solution, although strongly coloured, has a silent ECD spectrum being monomeric (*vide infra* Fig. 4a).

Such precise control of the nanomorphology for one and the same self-assembling tecton is rare. Clearly, this control is dependent on the final solvent mixture polarity and order of addition of the reagents. It has been previously shown that while the use of **FF** leads to tubes, the use of the closely related phenylglycine–phenylglycine leads to formation of nanospheres^28^. In this case it is not surprising that by slightly modifying the molecular tecton, one can arrive at very different self-assemblies at the micrometre scale.

This method of self-assembling tectons relies on dissolving the system in a minimal amount of a ‘good solvent' and injecting this into a much larger volume of ‘bad' solvent(s) in which the constructs become poorly soluble (Supplementary Fig. 22). The usual operating procedures for the formation of **FF** assemblies involve first physically dissolving the peptide powders into the good solvent that allows the **FF** building blocks to remain in their monomeric state, most often being HFIP or DMSO, while in this work dichloromethane was used, followed by the addition of a solvent that induces self-assembly (implicitly the bad solvent). This process was also referred to as ‘solvent switch'^20^. Temperature or pH switches have also been employed for inducing self-assembly^29^. The first use of this method was applied for BChls^42^ or their synthetic mimics^43^, and more recently to water-soluble porphyrins^44^,^45^, and even very recently for the parent **FF** dipeptide^46^. Moreover, a detailed study of the self-assembly of the tecton **Boc-F**_*L*_**F**_*L*_ initially dissolved into methanol and further diluted in water was also recently carried out by Gazit and co-workers^47^. Depending on the ratio of the two solvents and the concentration of **Boc-F**_*L*_**F**_*L*_, self-assembly into spheres that further evolve into fibrils and ultimately tubes has been observed. The process has been attributed to Ostwald-ripening processes of the nanospheres, leading to tubes as the most thermodynamically stable form^47^. However, in our study, no tubes have been observed. To better ascertain the three-dimensional architecture of the self-assemblies, we also imaged them with SEM following a critical point drying procedure and again, fibrillary, and not tubular morphologies were observed (Fig. 2).

### Electronic circular dichroism studies

**Figure 3:**
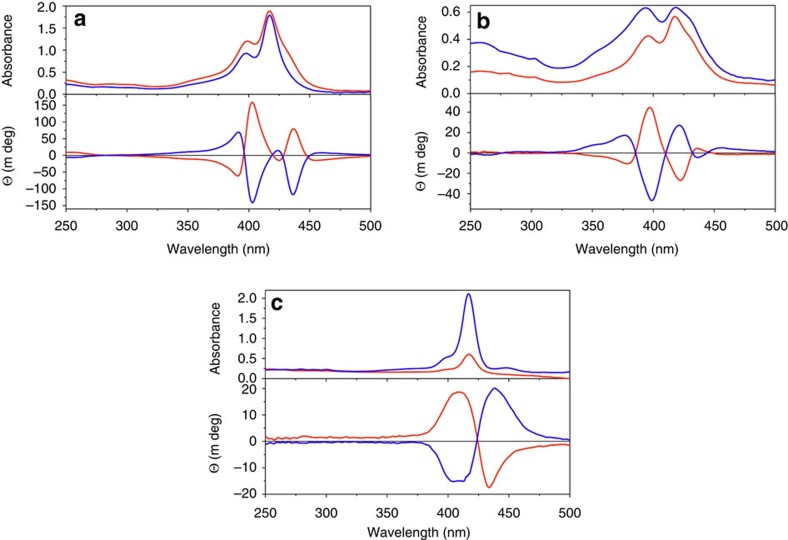
Ultraviolet-Vis absorption and ECD spectra. Blue traces are from the natural dipeptide conjugate **X-F**_*L*_**F**_*L*_**-TPP** while red traces are from **X-F**_*D*_**F**_*D*_**-TPP** with **X=Boc** (**a**) and **X=Fmoc** (**b**,**c**). (**a**) Self-assemblies obtained by injecting 3 μl of **Boc-FF-TPP** from a stock solution (2.8 mg dissolved in 350 μl dry dichloromethane) into dry *n*-heptane (400 μl). (**b**) Self-assemblies obtained by injecting 3 μl of **Fmoc-FF-TPP** from a stock solution (2.8 mg dissolved in 330 μl dry dichloromethane) into dry *n*-heptane (400 μl). (**c**) Film on the cuvette walls from the same cuvette as in **b** after decanting the solution. Supplementary Fig. 20 presents the corresponding spectra of unprotected **FF-TPP**.

**Figure 4:**
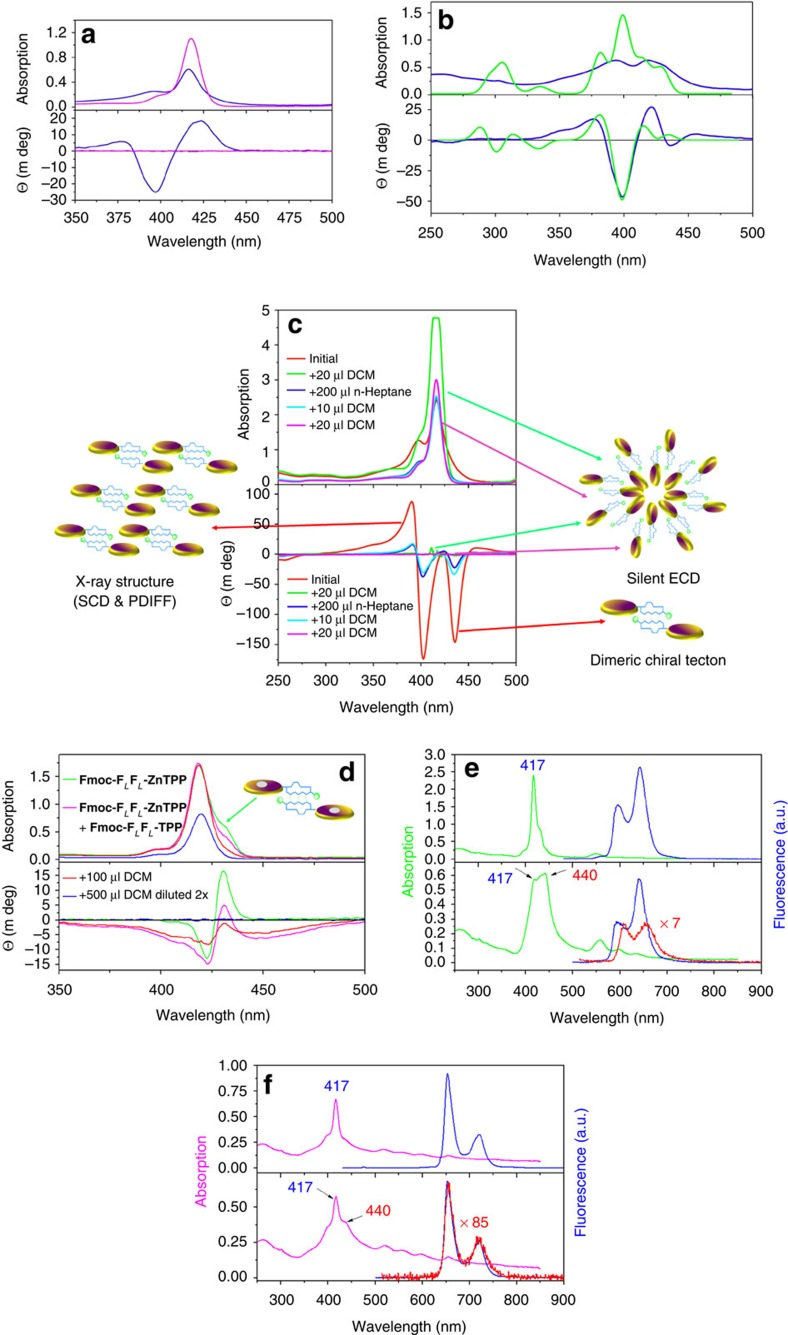
Ultraviolet-Vis absorption with ECD and fluorescence spectra of X-F_*L*_F_*L*_-TPP. (**a**) **Fmoc-F**_*L*_**F**_*L*_-**TPP** dissolved in dichloromethane (magenta traces with silent ECD spectrum in the Soret region) and after injection into *n*-heptane (blue traces). (**b**) Experimental (blue traces) versus calculated spectra (green traces) for self-assembled **Fmoc-F**_*L*_**F**_*L*_-**TPP** in *n*-heptane. An arbitrary scaling was performed at the maximum of the largest negative Cotton effect. (**c**) **Boc-F**_*L*_**F**_*L*_-**TPP** on changing the solvent composition as indicated in the inset. Half of the solution volume was removed from the cuvette and the corresponding amount of *n*-heptane was added. The Soret band of the green trace after addition of the first aliquot of 20 μl dichloromethane is slightly truncated due to saturation of the detector. The cartoons symbolize different self-assemblies of **TPP** (discs) via triple hydrogen bonding of the **FF-**dipeptide moieties (blue forms) protected by **Fmoc** or **Boc** groups (green spheres). (**d**) **Fmoc-F**_*L*_**F**_*L*_-**ZnTPP** after injection in *n*-heptane (green trace). In the chiral dimer, we presumed that due to the Fmoc protecting group there is no coordination to the Zn atom (grey disk). The magenta trace was obtained from **Fmoc-F**_*L*_**F**_*L*_-**ZnTPP** (1.85 mg dissolved in 400 μl dry dichloromethane) admixed (4/1, v/v) with **Fmoc-F**_*L*_**F**_*L*_-**TPP** (2.57 mg dissolved in 500 μl dry dichloromethane). A volume of 1.5 μl of this mixture was injected into *n*-heptane. Dilution with aliquots of dry dichloromethane gradually leads to a silent ECD (blue trace, after a twofold dilution). (**e**) Absorption of **Fmoc-F**_*L*_**F**_*L*_-**ZnTPP** after injection in *n*-heptane (green trace, lower panel) and after addition of dichloromethane (upper panel). The corresponding fluorescence spectra were obtained after excitation at 417 nm (blue traces) or at 440 nm (red trace). (**f**) Absorption of **Fmoc-F**_*L*_**F**_*L*_-**TPP** after injection in *n*-heptane (magenta trace, lower panel) and after addition of dichloromethane (upper panel). The corresponding fluorescence spectra were obtained after excitation at 417 nm (blue traces) or at 440 nm (red trace). The red fluorescence traces were vertically scaled by multiplying with factors of 7 and 85, which reflect the amount of quenching in the nonpolar solvent.

Figure 3a–c shows the absorption and ECD spectra within the Soret region for the **X-FF-TPP** conjugates with an X N-terminal Boc or Fmoc protecting group. After self-assembly, very intense and multiple Cotton effects prove that the porphyrin chromophores are excitonically coupled^40^. When two or more chromophores are in proximity in a chiral environment, their transition dipole moments can couple to give a characteristic split in the ECD signals. The exciton chirality method is well established with porphyrins as reporter groups^48^ and can be used to infer the absolute configuration of such constructs from the sign of the excitonic couplet. It has been previously demonstrated that in self-assembled chromophores, after excitation by visible light, the resulting excitons are long-lived and can travel extended distances exceeding 20 nm (ref. 8). Depending on the Boc or Fmoc protecting groups, there are some differences in the amplitudes but not in the signs of the multiple Cotton effects as detailed in Supplementary Fig. 21. Thus for the Fmoc derivative the most intense negative Cotton effect is at 398 nm while the longest wavelength Cotton effect at 437 nm has only a minimal negative value. The Boc derivative has almost equally strong negative Cotton effects at 403 and 437 nm. Also the absorption maxima of some bands, as seen from the ultraviolet-Vis spectra, are minimally shifted by only 2 nm. From these differences we infer that although the overall architecture must be similar, a slightly different orientation of the porphyrin transition dipole moments must exist in the Boc- compared with the Fmoc-protected constructs. In the latter case, we also observed the formation of a thin film, which was fluorescent, on the walls of the cuvette. In the film, the absorption maxima are shifted to 425 nm which corresponds to a zero crossing of the Cotton effects in the ECD spectra (Fig. 3c). This is again typical of *J*-aggregates and the arrangement of the porphyrins within the film is very different from the species in solution. The unprotected **FF-TPP** self-assembles and also forms, although less readily, a thin film whose ECD contribution dominates even the solution spectra (Supplementary Fig. 17). These films give a strong bisignate signal proving again strong exciton coupling. The **Fmoc-F**_*L*_**F**_*L*_**-TPP** films exhibit a positive exciton couplet (that is, positive Cotton effect at the longer wavelength) while the film of **Fmoc-F**_*D*_**F**_*D*_**-TPP** gives a perfectly mirror imaged negative couplet. We could reproduce well both the absorption and ECD film spectra by calculations performed on a dodecamer of **Boc-F**_*L*_**F**_*L*_**-TPP** having the porphyrin rings in register and double hydrogen bonding between the **FF** moieties as shown in Supplementary Fig. 30. The solutions in pure dichloromethane have a silent ECD spectrum in spite of their strong Soret absorption band (Fig. 4a). This is due to the negligible chiral perturbation of the porphyrin chromophore by the **FF-**dipeptide when in a solvated monomeric state. However, upon injection into *n-*heptane, because the basic building block is a chiral head-to-head dimer, there is a strong excitonic coupling between the porphyrins which results in multiple (at least trisignate) Cotton effects (Fig. 4a). This ECD signature could again be confidently reproduced by theoretical calculations on such a dimer whose exact geometry could be inferred from powder and single crystal X-ray diffraction analyses (*vide infra*).

By adding small aliquots of dichloromethane, the solvated self-assemblies change their morphology gradually (Fig. 2), until an almost silent ECD spectrum is obtained (Fig. 4c). Remarkably, further addition of *n*-heptane, restores the exciton couplets. This is, to our knowledge, the first artificial antenna system which can be switched reversibly between different morphologies. This provides an on/off switching effect of the exciton coupling and therefore of the inherent antenna function.

### Fluorescence studies

We investigated if the self-assemblies can function as an antenna by stationary and time-resolved fluorescence measurements (Supplementary Fig. 23). In the fibrillary aggregates, the fluorescence decay is shortened under the 20 ps time resolution of our experimental setup. However, albeit strongly, it is clearly not completely quenched. The lifetime of these fibrillary assemblies is much shorter in comparison with that of TPP in the monomeric state which is 9.0–12.8 ns depending on the solvent and which is similar to the fluorescence lifetime measured for the nanospheres that show a much longer mono-exponential decay of 9.3 ns (ref. 37). Because the fibrils are composed of densely packed chromophores, as inferred from the microscopy images and the X-ray data (*vide infra*), non-radiative decay pathways and concentration quenching must operate.

Furthermore, we metallated with zinc acetate the free base **Fmoc-F**_*L*_**F**_*L*_**-TPP** and obtained the corresponding **Fmoc-F**_*L*_**F**_*L*_**-ZnTPP** with modified absorption and emission wavelengths. We expected that the Fmoc protecting group will prevent coordination of the central Zn atom by the terminal amino group and thus let the FF dipeptide dictate the self-assembly algorithm in a similar manner as for the free base, although the π–π interactions between the porphyrin units might differ. Fluoresence quantum yields in dichloromethane solution, where the species are disassembled and give silent ECD spectra as the porphyrin transitions are not chiraly perturbed, were for the free base **Fmoc-F**_*L*_**F**_*L*_**-TPP** 10.8% and only 2.7% for **Fmoc-F**_*L*_**F**_*L*_**-ZnTPP**. The quantum yields were determined from corrected emission spectra using the relative method and having **TPP** and **ZnTPP** as standards with Φ=11% and 3%, respectively^49^. In the ultraviolet-Vis and ECD spectra, upon self-assembly of the Zn-analog, similar broad and red-shifted maxima with intense Cotton effects were encountered (Fig. 4d–f). The fluorescence is intense when exciting at 417 nm in the Soret band corresponding to monomeric species (Fig. 4e, blue traces) but quenched by a factor of seven when exciting in the shoulder at 440 nm (Fig. 4e red trace). For the corresponding free base, the quenching in the fibrillary state is even more intense by a factor of 85 (Fig. 4f, red trace).

We were unable to put into evidence energy transfer within self-assemblies of **Fmoc-F**_*L*_**F**_*L*_**-ZnTPP** admixed with various amounts (1:10, 1:4 or 1:1) of the free base **Fmoc-F**_*L*_**F**_*L*_**-TPP** (data not shown). We expected to observe an enhanced quenching of the energy donor **ZnTPP** emission at ∼600 nm with a corresponding increase in the free base acceptor at 720 nm as encountered in previous studies^50^. Alternatively, in the fluorescence excitation spectra monitored at 720 nm, where exclusively **Fmoc-F**_*L*_**F**_*L*_**-TPP** emits, we could not distinguish between the absorption spectra of the Zn-donor and free base acceptor in a monomeric state. A possible explanation, which warrants further investigation, is whether the two units form segregated self-assemblies, in which energy transfer is precluded, due to larger than 10 nm distances between the zinc donor and free base porphyrin acceptor.

### FT-IR and powder X-ray diffraction studies

**Figure 5:**
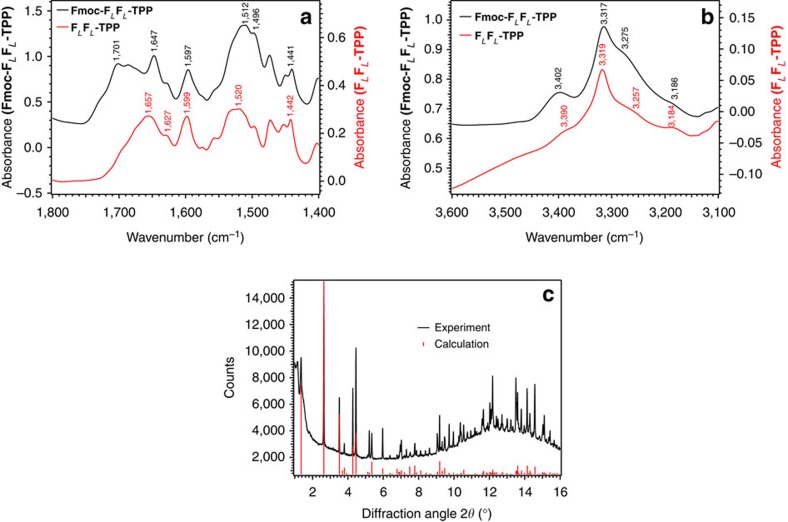
FT-IR spectra and XRD powder diffraction of Fmoc-F_*L*_F_*L*_-TPP microcrystals. FT-IR spectra of **F**_*L*_**F**_*L*_**-TPP** dipeptide KBr pellets in the spectral region of (**a**) CO, CN, CC and (**b**) NH stretching vibrations. The black trace is from the Fmoc-protected **TPP-F**_*L*_**F**_*L*_ dipeptide while the red trace is from the unprotected **F**_*L*_**F**_*L*_-**TPP** dipeptide. XRD powder pattern of the same **Fmoc-F**_*L*_**F**_*L*_**-TPP** microcrystals (**c**). The black trace is the experimental powder diffraction pattern (*λ*=1.0000 Å). Red lines are the simulated reflections from the single-crystal data described below. A logarithmic scaling of the measured intensities as a function of 2Θ is presented in Supplementary Fig. 31.

Crystallization of **FF-**dipeptides to give single crystals suitable for X-ray diffraction studies is challenging^23^. From various crystallization trials using the **Fmoc-F**_*L*_**F**_*L*_-**TPP** dissolved in dry dichloromethane which was layered with a nonpolar solvent such as *n-*hexane, cyclohexane or *n-*heptane and allowing a slow diffusion process to occur, we repeatedly obtained fibrillary crystals that were long but very thin. Using material from the same crystallization vial, we could both measure FT-IR spectra (Fig. 5a,b) and also obtain high-quality powder X-ray diffraction data at the PDIFF beamline of the ANKA synchrotron, Karlsruhe, which showed that the material had a high degree of crystallinity (Fig. 5c).

From the FT-IR data, it can be inferred from the low frequencies of both the C=O and N–H stretching vibrations that intermolecular hydrogen bonding of type N-H···O=C< must occur within the crystal. Comparison of the spectra of **Fmoc-F**_*L*_**F**_*L*_-**TPP** with the unprotected **F**_*L*_**F**_*L*_-**TPP** (Fig. 5a) clearly shows the additional band due to the Fmoc ester carbonyl at a position around 1,700 cm^−1^, a large downshift in comparison with the value of ∼1,740 cm^−1^ to be expected if no hydrogen bonding was occurring^51^. The amide I band with the C=O frequency at 1,647 cm^−1^ in the Fmoc derivative is consistent with hydrogen bonding, supported by the fact that this band was at 1,657 cm^−1^ and somewhat broader in the free base. The porphyrin breathing mode near 1,600 cm^−1^ was unaffected by linking the Fmoc moiety.

In the N–H stretching region (Fig. 5b), the sharp band near 3,318 cm^−1^ in both compounds can clearly be assigned to the N–H groups of the TPP moiety. Other readily assignable N–H frequencies include the amide B band near 3,185 cm^−1^, present in both compounds, the band at 3,257 cm^−1^ present only in unprotected **F**_*L*_**F**_*L*_-**TPP** and therefore identifiable as the symmetrical stretching of the NH_2_ group, and the band at 3,275 cm^−1^ present only in **Fmoc-F**_*L*_**F**_*L*_-**TPP** and therefore identifiable as the N–H stretching of the primary amine formed on attachment of the Fmoc group. Further assignments, in particular for the amide A N–H stretching bands of the dipeptide, are difficult because of band overlap and would be speculative without further investigations. For the present work, it suffices to note that all the bands observed are in the spectral region characteristic for N–H groups functioning as hydrogen bond donors, while no bands occur in the range above 3,450 cm^−1^ where N–H groups without hydrogen bonds would be expected to appear^51^. It is also interesting to note the enhanced amplitude of the band near 3,400 cm^−1^ upon insertion of the Fmoc group, consistent with the large increase in intensity of the amide A band on hydrogen bonding, as predicted by *ab initio* calculations^52^. Supplementary Fig. 24 presents a picture of a slide used for the FT-IR measurements on the microscope while Supplementary Figs 25 and 26 present additional FT-IR spectra.

### Single-crystal X-ray diffraction studies

Encouraged by the powder diffraction results, perseverance over a period of 3 years in growing single crystals by setting up crystallization tubes with various solvents and concentrations eventually resulted in single crystals that were just large enough for a single-crystal structure analysis. From one of these, a data set could be measured on beamline I19 at the Diamond Light Source, Harwell, UK. The data were of relatively low resolution with no diffraction observed beyond 1.1 Å, and that in the range 1.5–1.1 Å being weak. Significant radiation damage was observed so that a compromise between diffraction intensity and data set completeness was necessary. However, the data could be solved by direct methods and then refined isotropically with suitable restraints and constraints (Supplementary Methods). The structure obtained is fully adequate for the purposes of illustrating the general molecular conformation and the packing of the molecules in the solid state. The powder pattern simulated from the final single-crystal structure is in excellent agreement with the experimental data (Fig. 5c).

**Figure 6:**
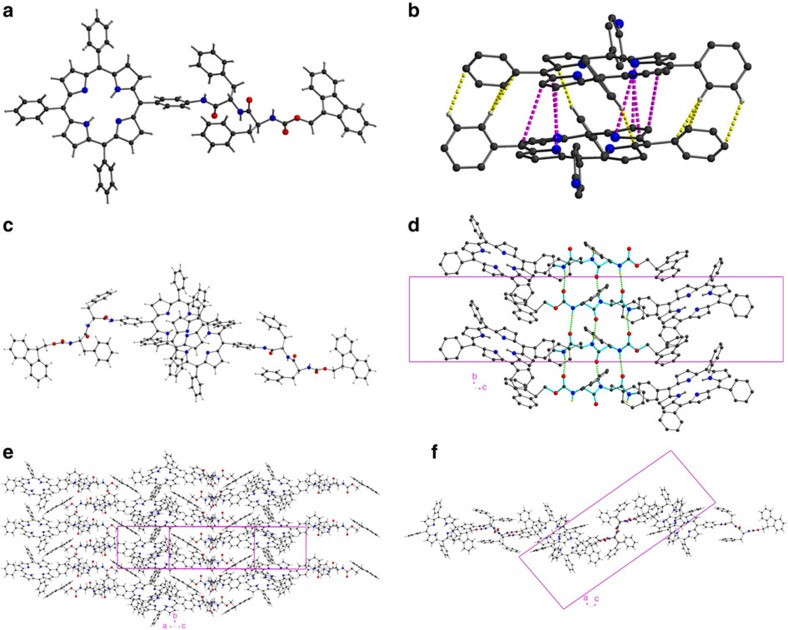
Single-crystal X-ray structure of Fmoc-F_*L*_F_*L*_-TPP. (**a**) Molecule 1. (**b**) Dimer formed between Molecules 1 and 2 with an enlargement of the two porphyrin moieties (their **Fmoc-F**_*L*_**F**_*L*_ units are omitted for clarity) showing C–H···C interactions in yellow and *π–π* interactions in purple. (**c**) Another view of this dimer, perpendicular to the porphyrin planes, showing the slipped face-to-face geometry typical to *π*-stacking in porphyrinic *J*-aggregates. (**d**) Intermolecular ‘β-pleated sheet' hydrogen bonding (shown as green dashed lines) between **-F**_*L*_**F**_*L*_**-** moieties (highlighted in cyan) forming stacks in the *b* direction. The shifts of the IR frequencies to lower wavenumbers observed in the FT-IR spectra are thus fully accounted for. (**e**) Interdigitation of porphyrin groups between the β-pleated stacks forming layers parallel to [102], viewed projected onto [102]. (**f**) as (**e**) but viewed down the *b*-axis. Other views are presented in Supplementary Figs 33 and 34 while a larger portion of the crystal lattice is shown in Supplementary Fig. 35.

The compound **Fmoc-F**_*L*_**F**_*L*_**-TPP** crystallizes in the space group *P*2_1_ with Z=4 with two independent but isostructural molecules in the asymmetric unit. One of these is shown in Fig. 6a. The two molecules form a supramolecular dimer in the crystal (Fig. 6b,c) in which the mean planes of the two porphyrin rings are almost exactly co-parallel (dihedral angle 0.70°) and 3.684 Å apart, although a degree of puckering of the rings results in some C··C or C··N distances being as close as 3.40 Å. They are therefore more tightly packed than the (B)Chl special pairs of reaction centres which have shortest atom neighbours about 3.60 Å apart^12^. In addition to *π*···*π* interactions between the porphyrin rings, the three unsubstituted phenyl rings on each porphyrin are rotated by 50–59° out of their respective porphyrin plane, allowing additional C–H···*π* interactions (C··C 3.65–3.78 Å), which result in a slippage of the two rings relative to each other of ca. 3.31 Å.

The molecules are linked into stacks parallel to the *b* axis by hydrogen bonding between the **F**_*L*_**F**_*L*_ moieties. This is structurally similar to what is seen in protein β-pleated sheets and seems to be the dominant supramolecular interaction, which is fully consistent with the FT-IR data. The stacks form layers parallel to the [201] crystal plane (Fig. 6d) via interdigitation of the porphyrin rings. This interdigitation is not symmetrical; within a stack, adjacent porphyrin groups from different dimers have a significantly larger interplanar separation (*π–π* interactions all with C···C over 4 Å) and also a greater degree of slippage. The supramolecular interdimer interactions are therefore now only mediated by C–H···*π* interactions, with the shortest such C···C distance 3.44 Å (Fig. 6e).

Within these layers, the Fmoc groups are oriented with their normal axis parallel to the plane and can be seen edge-on on viewing the layer perpendicular this plane (Fig. 6e, and Supplementary Fig. 34). In this way, these Fmoc groups are well placed to mediate the third dimension of the crystal packing provided by *π*···*π* interactions between the Fmoc and porphyrin ring systems (minimum C···C 3.27 Å) linking the layers to give the three-dimensional packing (Supplementary Fig. 34). Viewed down the *b* axis, the overall packing is seen to be made up of layers oriented parallel to [001], composed of porphyrin and Fmoc groups, which are then separated by ‘β-pleated sheets' of **F**_*L*_**F**_*L*_ spacers. The remaining voids between the layers are occupied by the phenylalanine phenyl groups, which are thus not involved in any supramolecular interactions, and by lattice solvent molecules.

The extensive *π*-stacking that occurs between two adjacent porphyrin planes (minimum intermolecular C···C distance 3.45 Å) explains the red-shifted absorption spectra as well as the quenched fluorescence in comparison with the monomeric forms in polar solvents. Furthermore, by considering the geometry for the head-to-head dimer, TD-DFT calculations of the ECD spectrum (at the lc-wpbe/6–31 g(d) level of theory) gave a satisfactory correlation (shown in Fig. 4b) with the experimental spectrum. The positive–negative–positive triplet Cotton signature in the spectral region around 400 nm corresponds to the ECD fingerprint for excitonic couplings between the two split components of the Soret B band. In particular, the electronic transition polarized along the N–N direction of the porphyrin core is red-shifted (down to ∼435 nm) due to *J*-type interactions between the longitudinally displaced chromophores (that is, along the N–N axis), while *H*-aggregate-like interactions slightly blue shift the NH–NH polarized transition below 400 nm, giving rise to the twin peak in the absorption spectrum. Though the calculations reported here are for simple physical dimers, we expect a similar picture to hold in larger aggregates, with, in principle, larger shifts in the spectral positions for spatially more delocalized electronic excited states. The centre-of-mass delocalization extent of the exciton results from the subtle interplay between electronic interactions, coupling to vibrations, energetic and positional disorder. Supplementary Fig. 28 presents the calculated absorption spectrum of a **Boc-F**_*L*_**F**_*L*_**-TPP** monomer while Supplementary Fig. 29 shows drawings of the molecular orbitals and the corresponding transitions.

The crystal structure result explains perfectly the following observations (i) the amide I band position in the FT-IR spectra and the involvement of the Fmoc carbonyl group in hydrogen bonding, (ii) the red-shift in the electronic absorption spectra, (iii) the chiral dimeric assemblies giving rise to rise to a complex ECD signature, and (iv) the quenched fluorescence due to the rather close packing of the porphyrin rings which do not form tubular assemblies. The crystal structure is in line with what would be expected for the fibrillary assemblies, the crystallographic *a* axis being oriented along the fibre's length.

## Discussion

Natural light-harvesting systems are made from very labile chromophore complexes that under high illumination condition can be damaged. Besides using various photoprotection mechanisms, living organisms continuously renew the degraded components and recycle the metabolic important elements such Mg, Mn and Fe. The present study combined structural investigations on the supramolecular architectures of **F**_*L*_**F**_*L*_**-TPP** and a Zn-metallated derivative. We could show that this tecton, depending on the solvent's polarity, can form different assemblies. One of these has strongly coupled porphyrins which give a characteristic signature in their ECD spectra. Mirror-imaged Cotton effects were observed for the unnatural F_*D*_ analogues, revealing that the chirality is translated from the molecular level to the supermolecules. These chiral assemblies form fibrils and tangles, and could function as antenna systems having a large number of porphyrins units between which energy can be transferred efficiently over long distances to an energy trap, as is observed in natural light-harvesting systems. In the present case, the fluorescence is strongly quenched due to π-stacking of the porphyrin units, thus it would be only a poorly functioning antenna. This presents an inactive state where the radiant energy could be dissipated without it being funnelled to the trap. In solvents of higher polarity, the morphology changes abruptly to microspheres which have silent ECD spectra but much enhanced fluorescence. While such artificial systems having an incorporated energy sink have yet to be fabricated, the possibility to switch between active and passive states could be realized in microfluidic devices by pumping different solvent systems. FF-fibres are among the strongest of all-organic materials and their decoration with functional chromophores displaying high photostability could have applications for photovoltaics. The covalent coupling of the porphyrin moieties to such simple FF zippers conveys the advantage of permanently attached chromophores that do not risk leaching out of the system during assembly and disassembly cycles. This feature could enable decoupling at will of the antenna function in smart photovoltaic devices.

By combining spectroscopic methods with theoretical calculations we could infer the exact architecture of the fibrillary assemblies and these must be the same in the microcrystals obtained from low polarity solvents. X-Ray powder diffraction and single-crystal analyses finally converged to reveal a structure that matched the rather complex ECD signature. This type of analysis could be useful in elucidating the structure of assemblies which fail to give good quality single crystals amenable to X-ray diffraction studies. Alzheimer's amyloid fibrils, tangles and plaques provide further examples of notoriously difficult to crystallize peptides. Such aggregating peptides are long known to be associated with about 50 human protein misfolding diseases^53^,^54^. By elucidating these types of structures, inhibitors could be devised, both for an early diagnostic, and for therapies.

While the present manuscript was under construction, derivatives of **F**-protoporphyrin IX were reported and their self-assemblies were also shown to give solvent-dependent nanostructures with chiralities dictated by **F**_*L*_ or **F**_*D*_ (ref. 55). Although no detailed structural evidence was presented in that study, by comparison of the ECD spectra we can infer similarities between their structures and those found in our **FF-TPP** assemblies presented here.

## Methods

### Syntheses

The yields of the optimized reaction steps are detailed in the Supplementary Fig. 36. All new compounds were fully characterized and their ^1^H- and ^13^C-NMR spectra are given as the Supplementary Figs 1–18 while the synthetic protocols are given in the Supplementary Methods.

### Scanning electron microscopy

The samples were prepared in glass vials. The peptide powder was dissolved in dichloromethane, vortex-mixed and diluted with *n*-heptane at a final concentration of 1 mM. For SEM observation, the samples were dried onto glass slides and sputter-coated with 15 nm thick gold (Baltec SCD 050). To evaluate the nature of the self-assembled elongated structures (hollow or not) a fixation and critical point drying procedure was additionally followed. The samples were fixed in a buffer (2% glutaraldehyde and 2% paraformaldehyde in 0.1 M sodium cacodylate) at pH 7.4 for half an hour at 4 °C, washed twice with the above-mentioned buffer, post fixed in 1% aqueous OsO_4_ for 30 min at 4 °C, and rinsed again as above. After fixation, the samples were dehydrated through a graded ethanol series, 30–50–70–90–100% at 4 °C, followed by dry alcohol at room temperature. Dehydrated samples were critical point dried (Baltec CPD 030) and mounted on stubs prior to sputter coating with 15 nm thick gold (Baltec SCD 050). Observation was carried out using a JEOL JSM-6390LV scanning electron microscope at 15 kV operating voltage.

### Crystallization trials and crystallography

Glass tubes of 5 mm internal diameter and 10 cm height were first filled to 8–10 mm with a concentrated CH_2_Cl_2_ solution of **Fmoc-F**_*L*_**F**_*L*_**-TPP**. Then another 8–10 mm of the tube were gently layered with a 1:1 mixture of CH_2_Cl_2_ and either dry *n-*heptane, hexane or cyclohexane after which the tube was completely filled with the respective nonpolar solvent and stoppered with a rubber septum. A typical crystal is pictured in Supplementary Fig. 32.

Powder diffraction data were measured on the PDIFF beamline at the ANKA synchrotron, Karlsruhe Institute of Technology, Germany, using Si-monochromated radiation of wavelength 1.000 Å and a Princeton 165 mm diameter CCD detector. The powder pattern was simulated from the single-crystal structural data using *Crystallographica*.

Single-crystal diffraction data were measured on the I19 beamline at the Diamond Light Source, Harwell, UK using Si-monochromated radiation of wavelength 0.6889 Å and a Rigaku Saturn 724+ CCD detector. The crystal, of dimensions 0.15 × 0.02 × 0.02 mm, was mounted on a Crystal Logic four-circle diffractometer and cooled to 100(2) K. A picture of a similar crystal is shown in Supplementary Fig. 32. In spite of the cooling, radiation damage was observed, and the data set obtained was the best compromise between data completeness and intensity before damage to the crystal became significant. The data were truncated at a resolution of 1.1 Å, although diffraction in the range 1.1–1.5 Å was mostly weak. The character of the structural determination therefore resembled more a ‘high-resolution protein structure' rather than a typical ‘small-molecule' analysis. The structure was solved using dual-space direct methods *SHELXT* (ref. 56) and refined using *SHELXL-2014* (ref. 57). In consequence of the limited resolution and quality of the data, a range of restraints and constraints were required, and atoms could only be refined isotropically. All H-atoms were placed in calculated positions using a riding model. Aromatic rings were constrained as rigid hexagons, and two C_4_N rings in the porphyrin moieties were constrained as regular pentagons. Planarity restraints were applied to some aromatic moieties. Geometrical similarity restraints were applied to many chemically related bond distances in the structure, and a few bond lengths were restrained to target values. One phenyl ring (C48A-C53A) undergoes high thermal motion and/or disorder—no disorder could be resolved, and the phenyl group could only be refined as a single ring of half-occupancy. The lattice cyclohexanes (two per porphyrin) could not be refined, and their contribution to the structure factors was calculated using *SQUEEZE*^58^.

As expected, the absolute configuration could not be determined from the Flack c parameter; the enantiomer was chosen that gave the correct configuration at the F_*L*_ chiral centres.

The CIF file for this structure is given as the Supplementary Data 1.

### FT-IR

Samples for FT-IR spectroscopy were prepared as KBr pellets. Typically, a sample of 1 mg was ground to homogeneity in a mortar together with 200 mg dry KBr (Sigma-Aldrich, Germany) and immediately pressed to a pellet (10 mm diameter) with 7.5 tons of pressure. Pellets were stored under dry nitrogen until measured. The infrared absorbance spectra of the KBr pellets were measured in transmission geometry under dry nitrogen purge in an Equinox 55 FT-IR spectrometer (Bruker, Germany) equipped with a liquid N_2_ cooled mercury cadmium telluride (MCT) detector in Karlsruhe. A pellet of pure KBr from the same batch was used as a reference for the absorbance measurements. Spectra were recorded as the average of 64 double-sided interferogram scans at 4 cm^−1^ resolution, giving a total acquisition time of 30 s. Fourier transformation of the interferograms to spectra was carried out with Blackman–Harris 3-term apodization, Mertz phase correction and non-linearity correction of the MCT signal. The spectra obtained were used without any further mathematical processing. Three separate measurements were carried out yielding identical spectra, one of which is shown in Fig. 5. Reproducibility was ascertained by measuring samples as similar KBr pellets on a Bruker Vertex 70 FT-IR spectrometer in Marseille (Supplementary Fig. 26).

### Electronic circular dichroism

A JASCO 815 spectrometer equipped with a Peltier unit assuring a constant temperature of 20.0±0.2 °C was used in Marseille. We employed rectangular Hellma quartz cuvettes having either 1 or 2 mm pathlengths. In Figs 3 and 4 the pathlength of the quartz cuvette was 2 mm. All spectra were baseline subtracted and recorded as one scan with 50 nm min^−1^. Solvents were thoroughly dried by storing over 3 Å molecular sieves.

### Stationary and time-resolved fluorescence

In Marseille stationary fluorescence spectra were measured with a JASCO FP-8600 fluorimeter equipped with a Peltier sample thermostat. For the time-resolved fluorescence spectra obtained in Karlsruhe we used as the excitation source a Ti:sapphire laser delivering sub-150 fs pulses with a repetition rate of 76 MHz and with a low excitation power of only 4–5 mW due to the very intense fluorescence observed on excitation in the Soret band.

### Computational details

Molecular mechanics and dynamics calculations were performed with the *Tinker* package^59^ in combination with the MM3 force-field^60^,^61^ to study the self-assembly of 12 **Boc-F**_*L*_**F**_*L*_**-TPP** molecules. Snapshots extracted from the MD trajectory were used to calculate the excitonic CD spectra of the stack^62^ (Supplementary Fig. 27) with electronic excited states calculated for each molecule of the stack at the INDO/SCI level^63^. The ECD spectrum for the head-to-head dimer was calculated using the geometry of the crystal structure with the Gaussian package^64^ at the lc-wpbe/6–31 g(d) level of theory. For additional computational studies see the Supplementary Methods Section.

### Data availability

Crystallographic data (excluding structure factors) for the structure in this paper have been deposited with the Cambridge Crystallographic Data Centre (CCDC), under deposition number CCDC 1415777. These data can be obtained free of charge from The Cambridge Crystallographic Data Centre via www.ccdc.cam.ac.uk/data_request/cif. All remaining data that support the findings of this study are available from the corresponding authors upon request.

## Additional information

**How to cite this article:** Charalambidis, G. *et al.* A switchable self-assembling and disassembling chiral system based on a porphyrin-substituted phenylalanine–phenylalanine motif. *Nat. Commun.* 7:12657 doi: 10.1038/ncomms12657 (2016).

## Supplementary Material

Supplementary Information Supplementary Figures 1-36, Supplementary Methods and Supplementary Reference.

Supplementary Data 1 CIF file associated with the single crystal structure determination of Fmoc-FF-TPP.
