# Supplementary material for: A switchable self-assembling and disassembling chiral system based on a porphyrin-substituted phenylalanine–phenylalanine motif
Source: Nat Commun. 2016 Sep 1;7:12657. doi: 10.1038/ncomms12657 (PMC5025786; doi:10.1038/ncomms12657)
Supplement: Supplementary Information — Supplementary Figures 1-36, Supplementary Methods and Supplementary Reference. [file ncomms12657-s1.pdf]

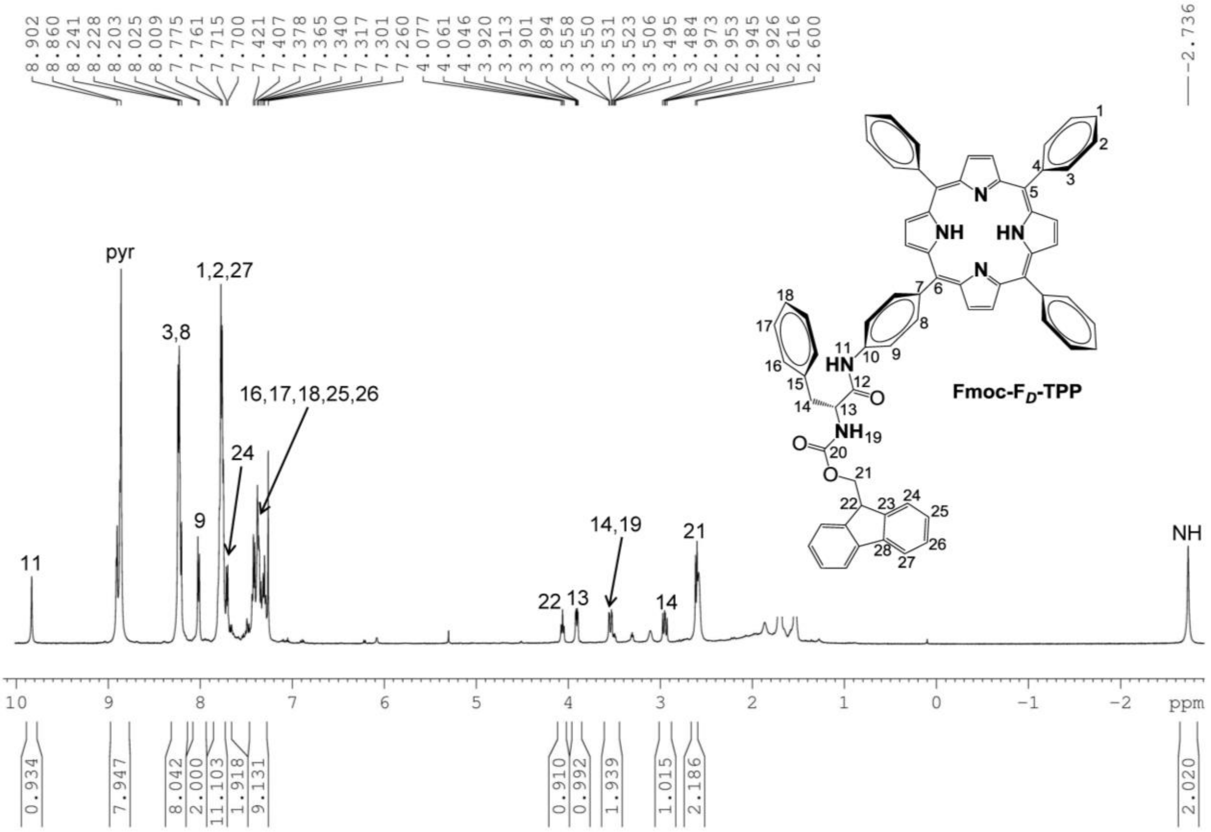

Supplementary Figure 1. ^{1}H NMR spectrum of compound **Fmoc-F$_{D}$-TPP** in $CDCl_3$.

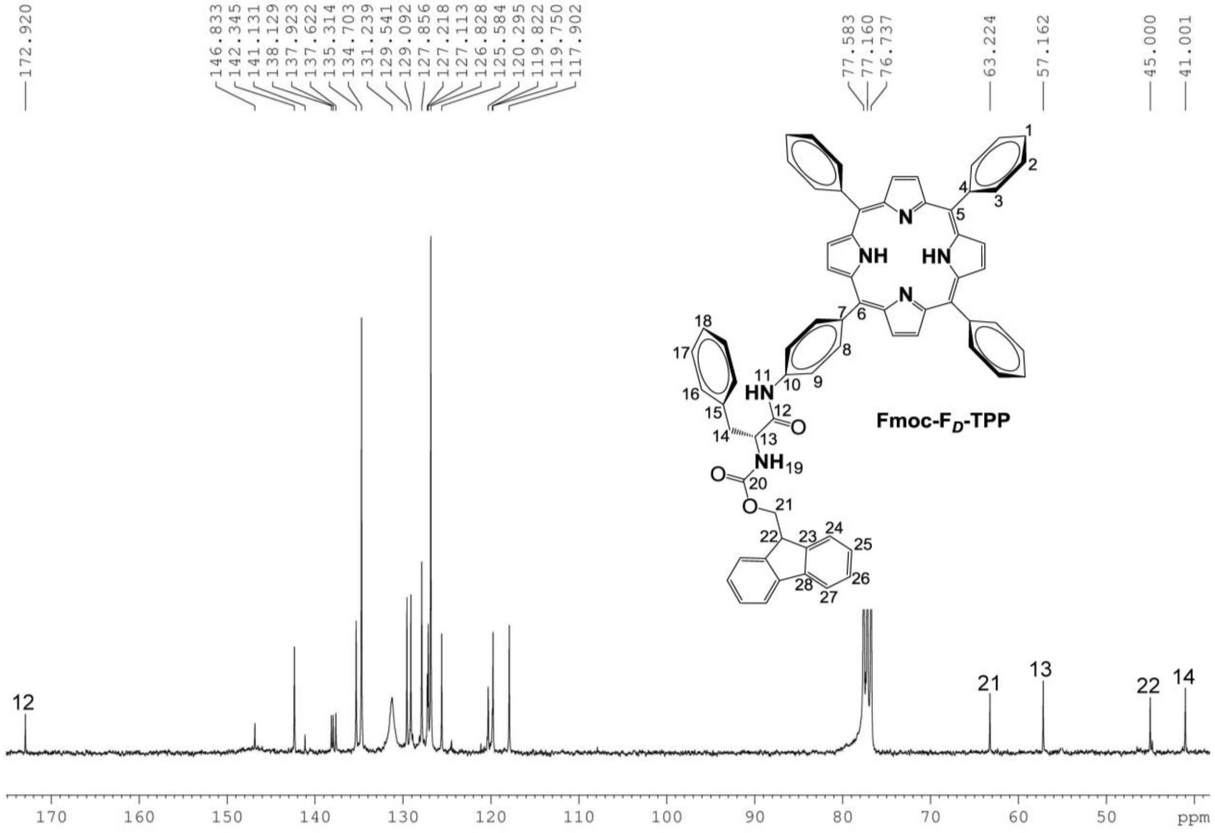

Supplementary Figure 2. ^{13}C NMR spectrum of compound **Fmoc-F$_{D}$-TPP** in $CDCl_3$.

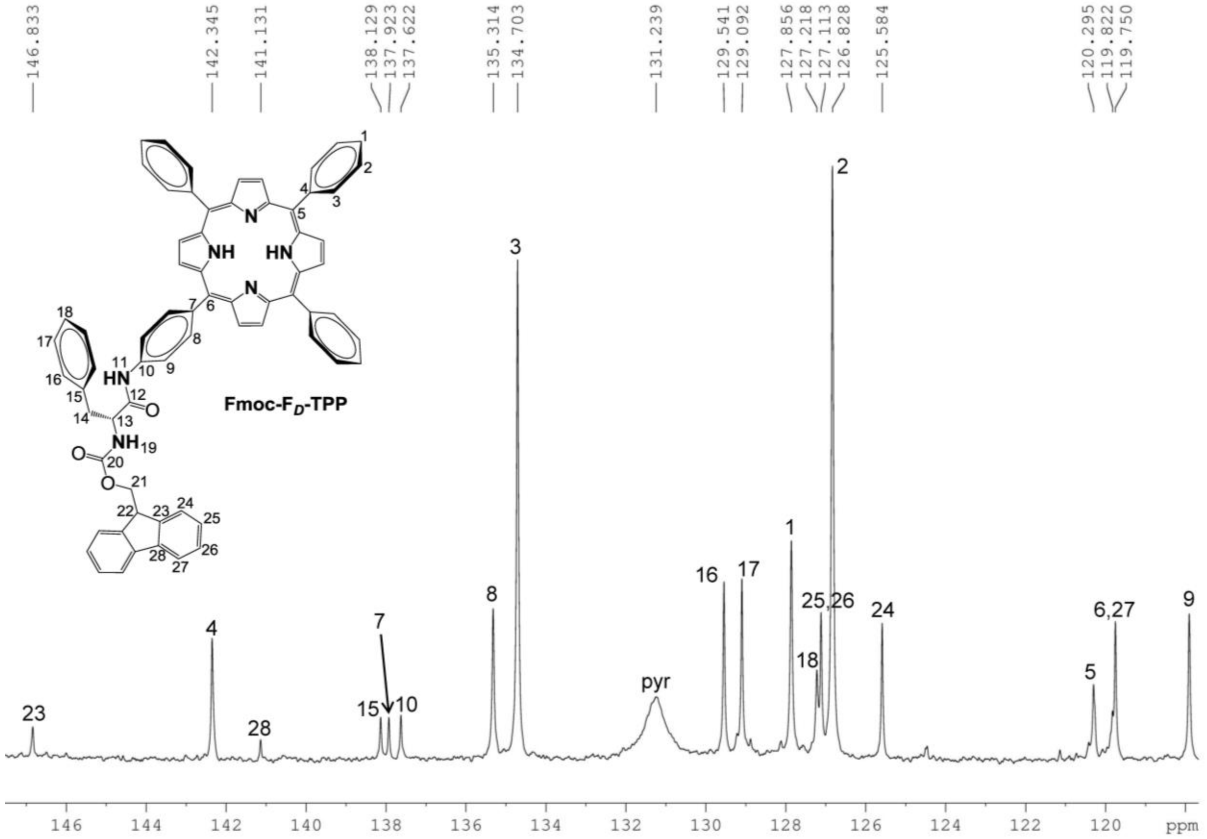

Supplementary Figure 3. Aromatic region of the ^{13}C NMR spectrum for compound **Fmoc-F*D*-TPP** in $CDCl_3$.

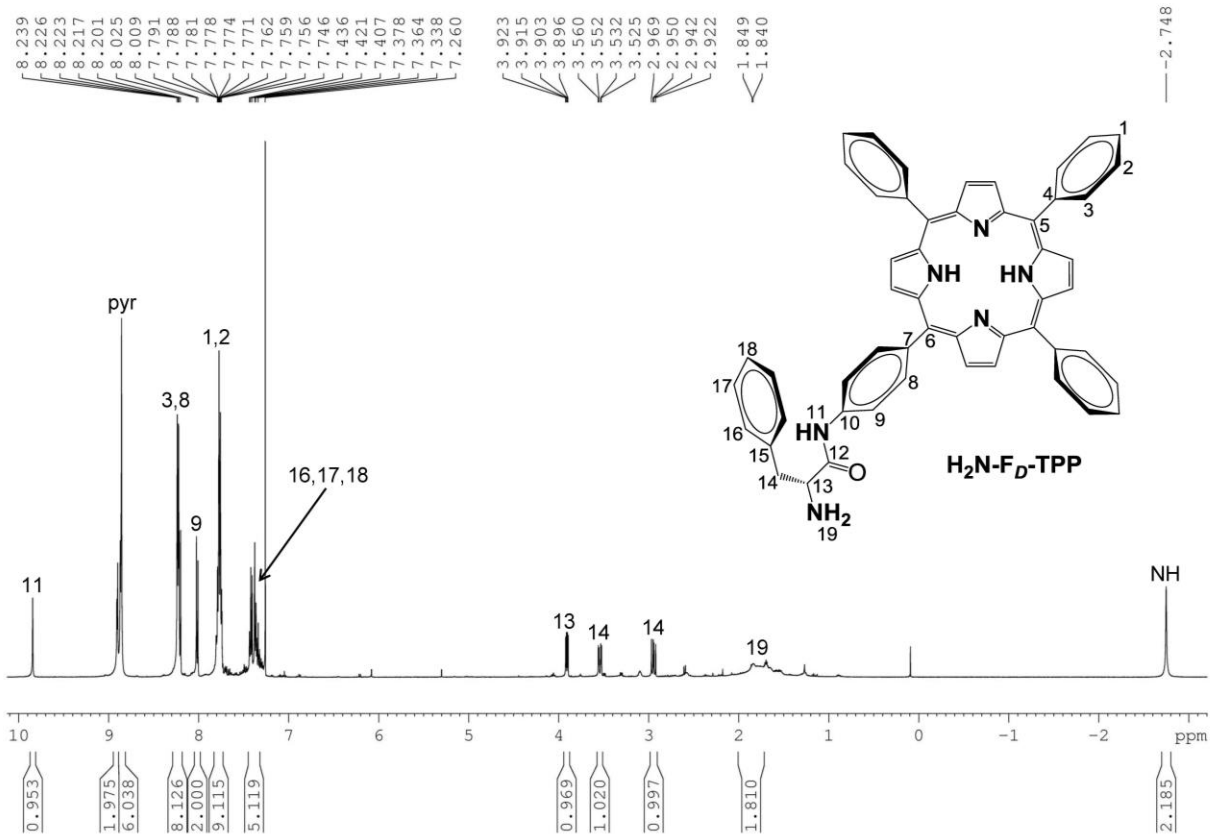

Supplementary Figure 4. ^{1}H NMR spectrum of compound **H_2N-F_D-TPP** in $CDCl_3$.

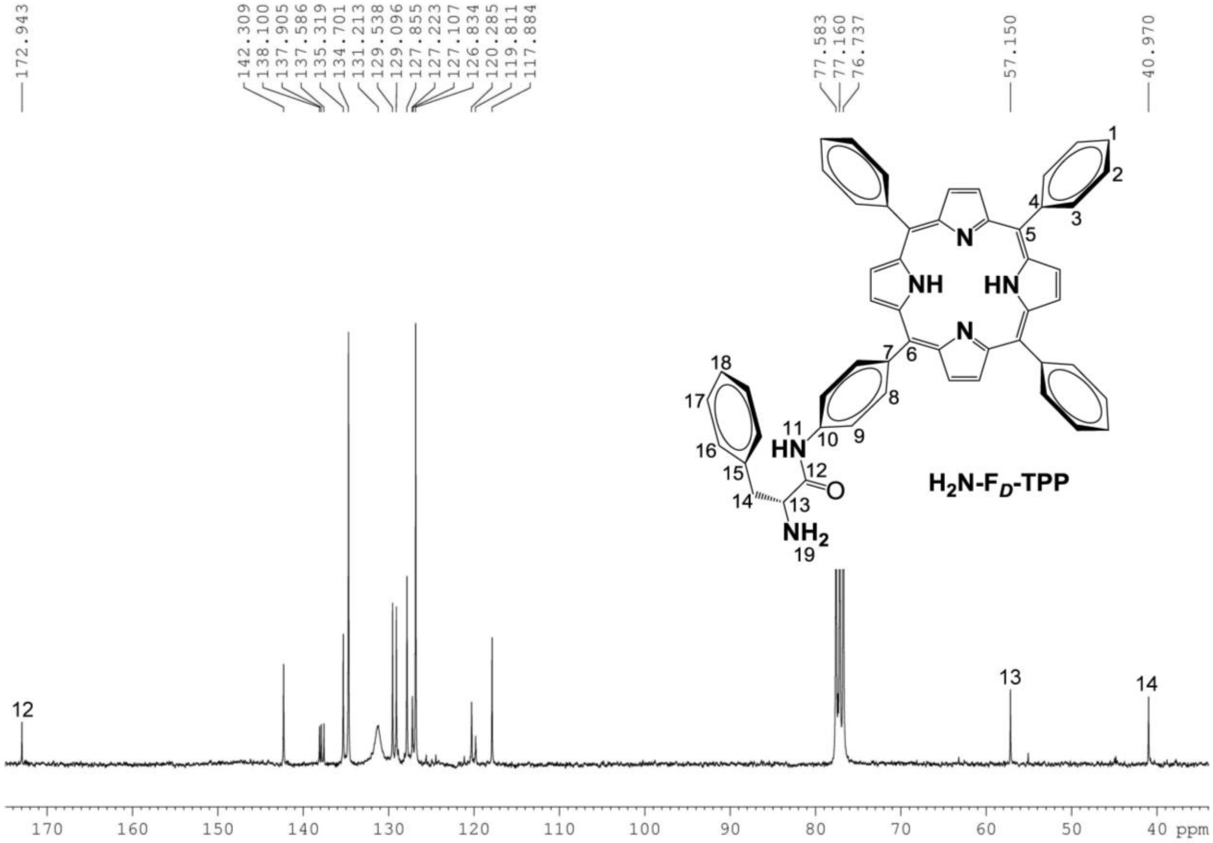

Supplementary Figure 5. ^{13}C NMR spectrum of compound **H_2N-F_D-TPP** in $CDCl_3$.

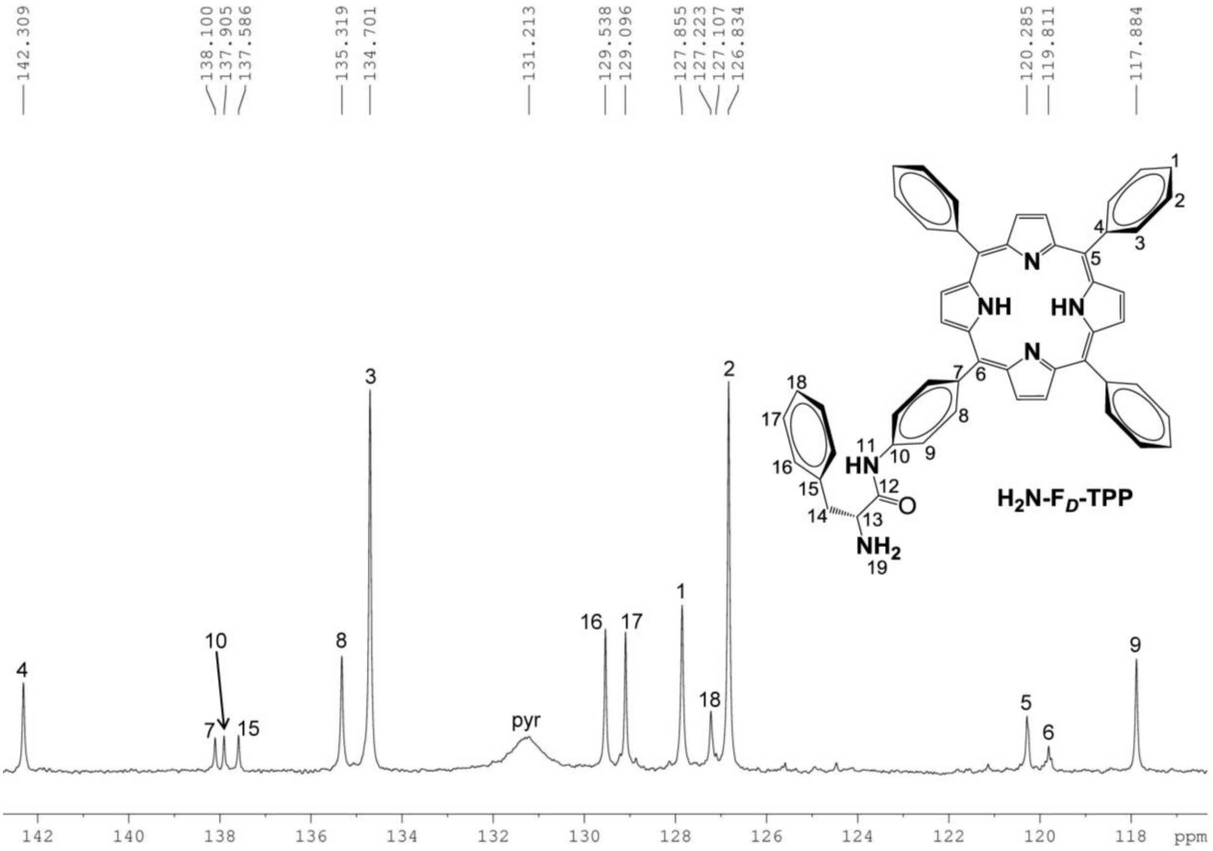

Supplementary Figure 6. Aromatic region of the ^{13}C NMR spectrum for compound **H_2N-F_D-TPP** in $CDCl_3$.

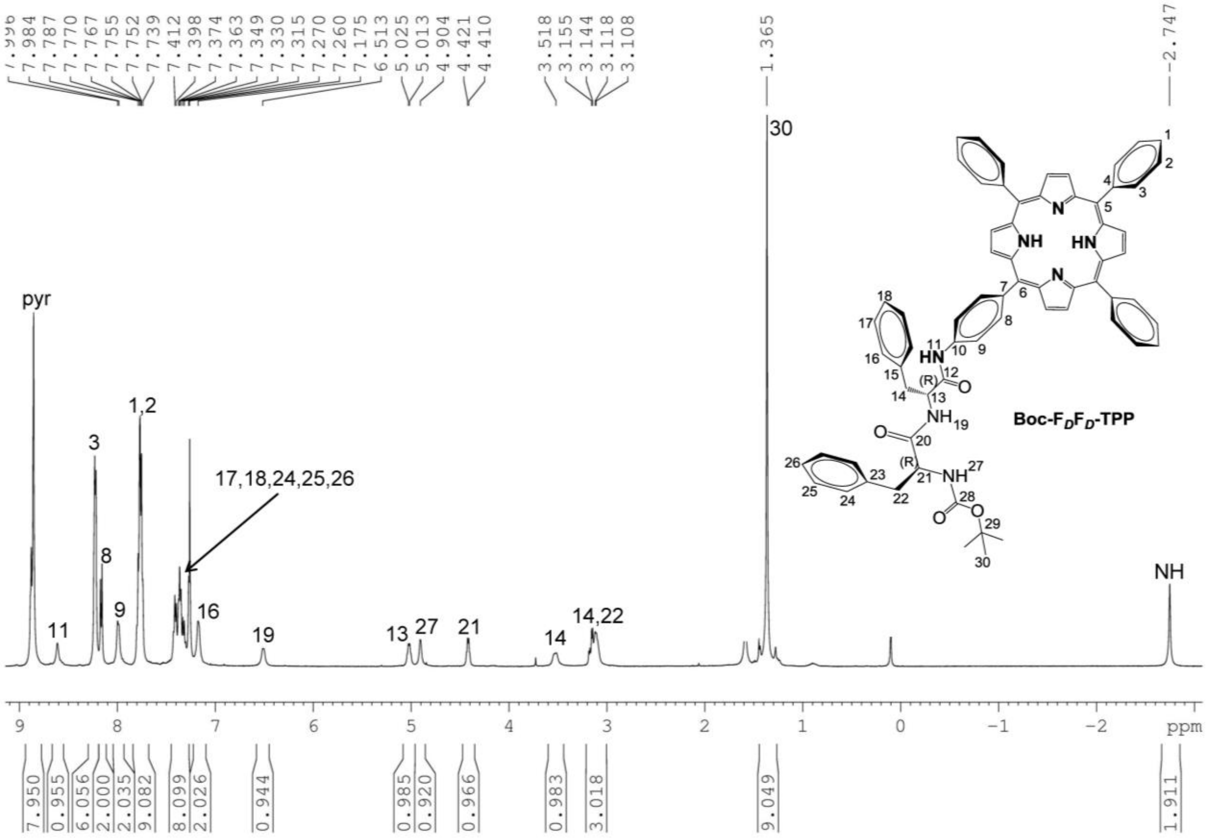

Supplementary Figure 7. ^{1}H NMR spectrum of compound **Boc-F$_D$F$_D$-TPP** in $CDCl_3$.

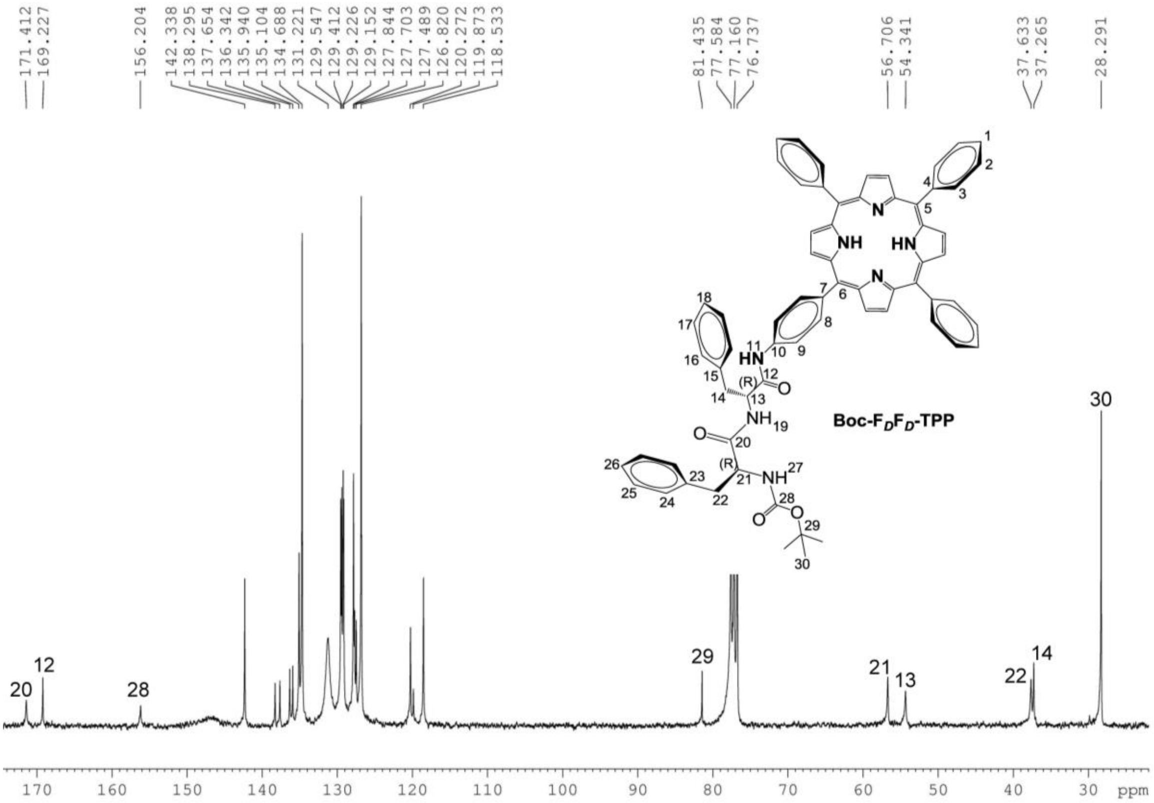

Supplementary Figure 8. ^{13}C NMR spectrum of compound **Boc-F$_D$F$_D$-TPP** in $CDCl_3$.

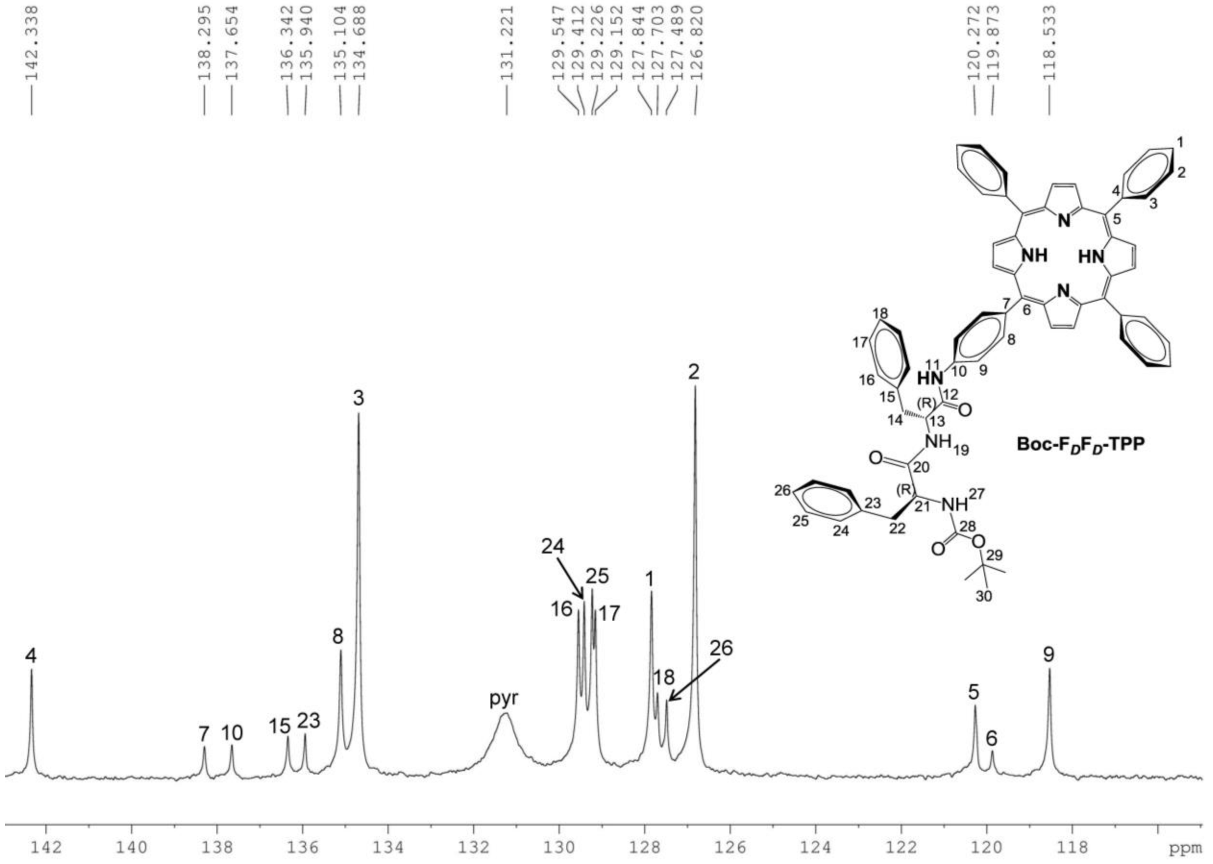

Supplementary Figure 9. Aromatic region of the ^{13}C NMR spectrum for compound **Boc-F_DF_D-TPP** in $CDCl_3$.

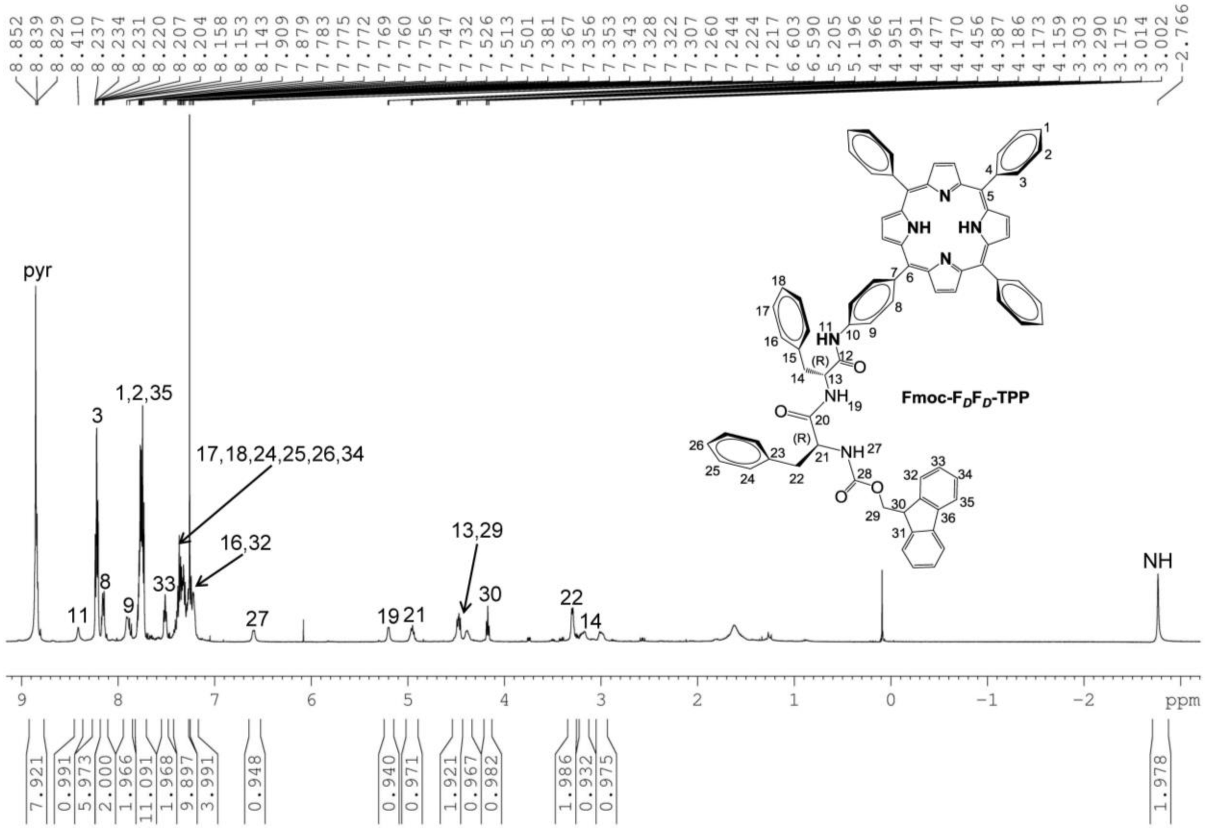

Supplementary Figure 10. ^{1}H NMR spectrum of compound **Fmoc-F*D*F*D*-TPP** in $CDCl_3$.

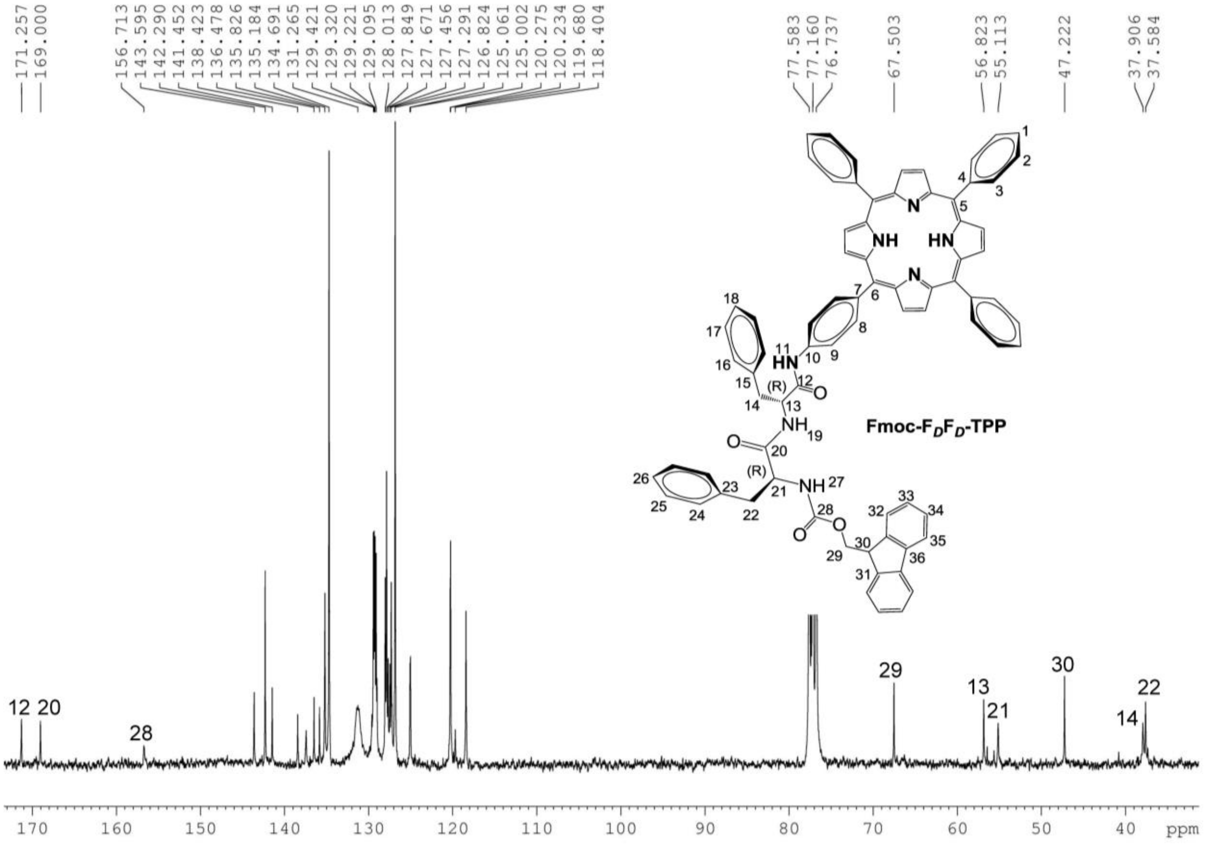

Supplementary Figure 11. ^{13}C NMR spectrum of compound **Fmoc-F*D*F*D*-TPP** in $CDCl_3$.

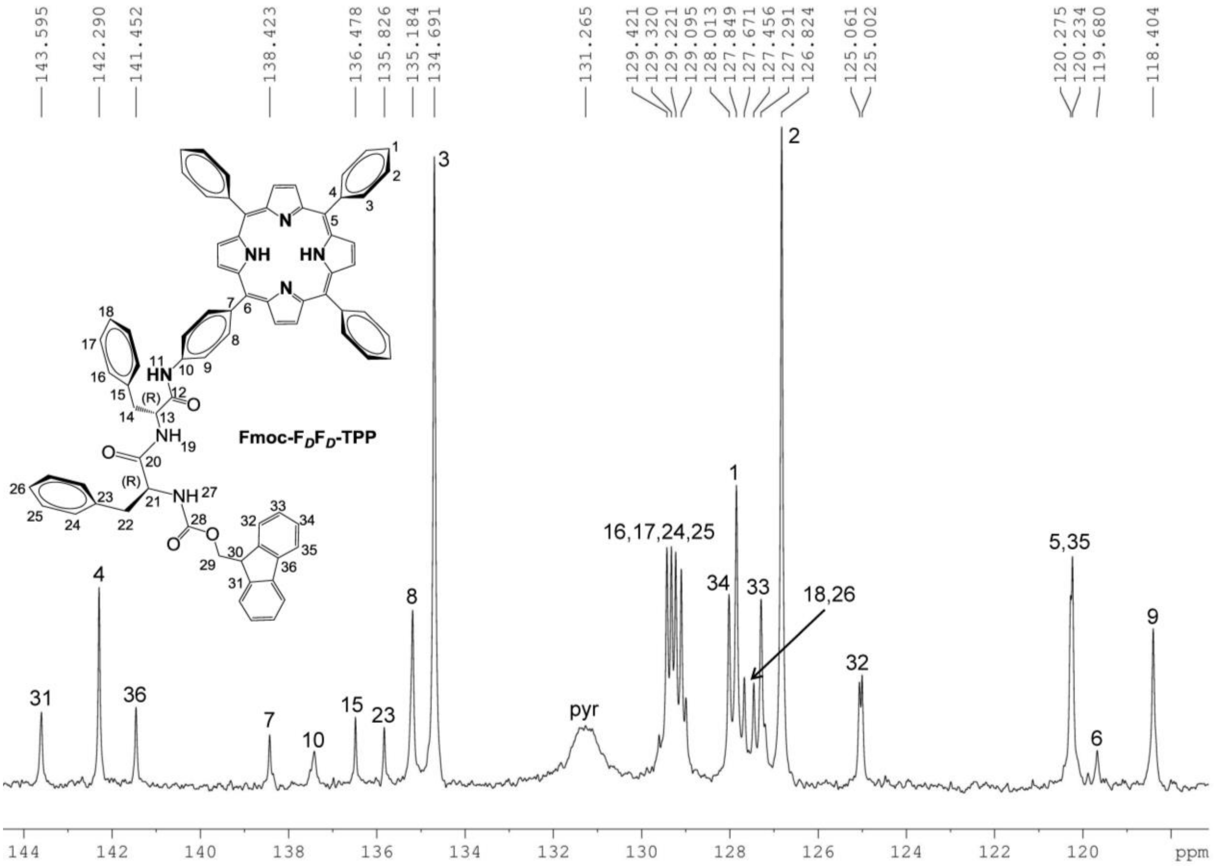

Supplementary Figure 12. Aromatic region of the ^{13}C NMR spectrum for compound **Fmoc-F$_D$F$_D$-TPP** in $CDCl_3$.

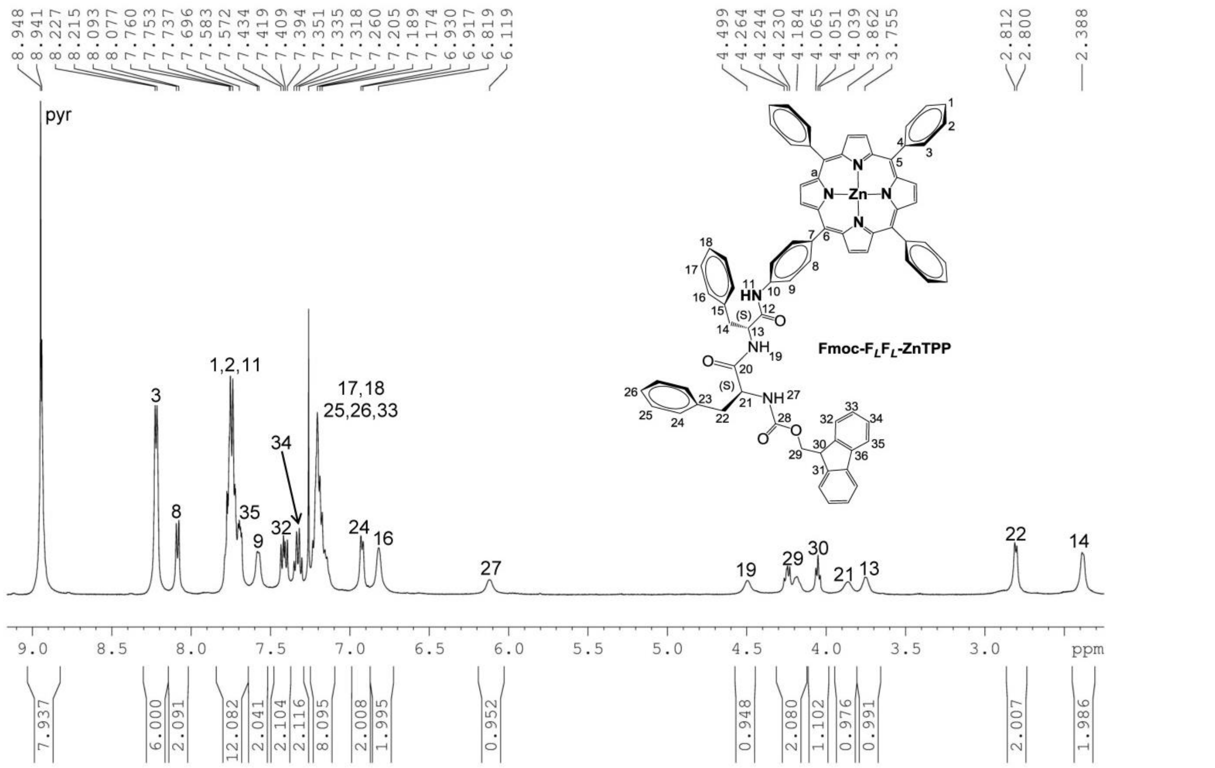

Supplementary Figure 13. ^{1}H NMR spectrum of compound **Fmoc-F$_L$F$_L$-ZnTPP** in $CDCl_3$.

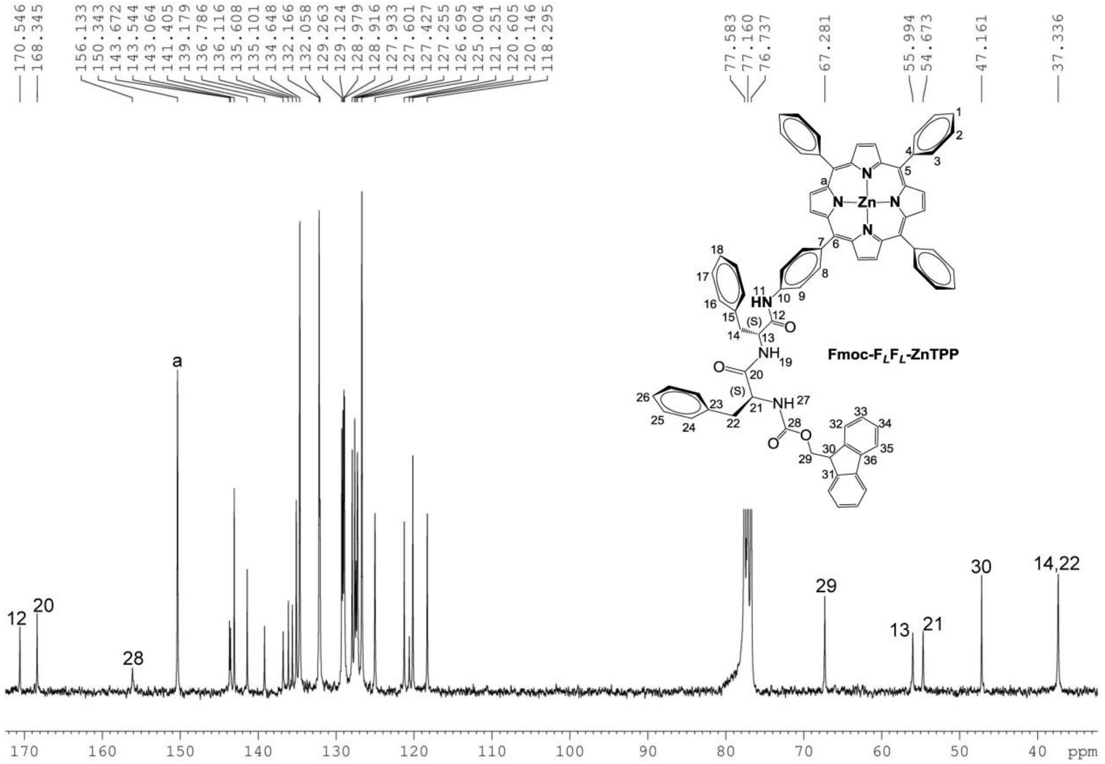

Supplementary Figure 14. ^{13}C-NMR spectrum of compound **Fmoc-F$_L$F$_L$-ZnTPP** in $CDCl_3$.

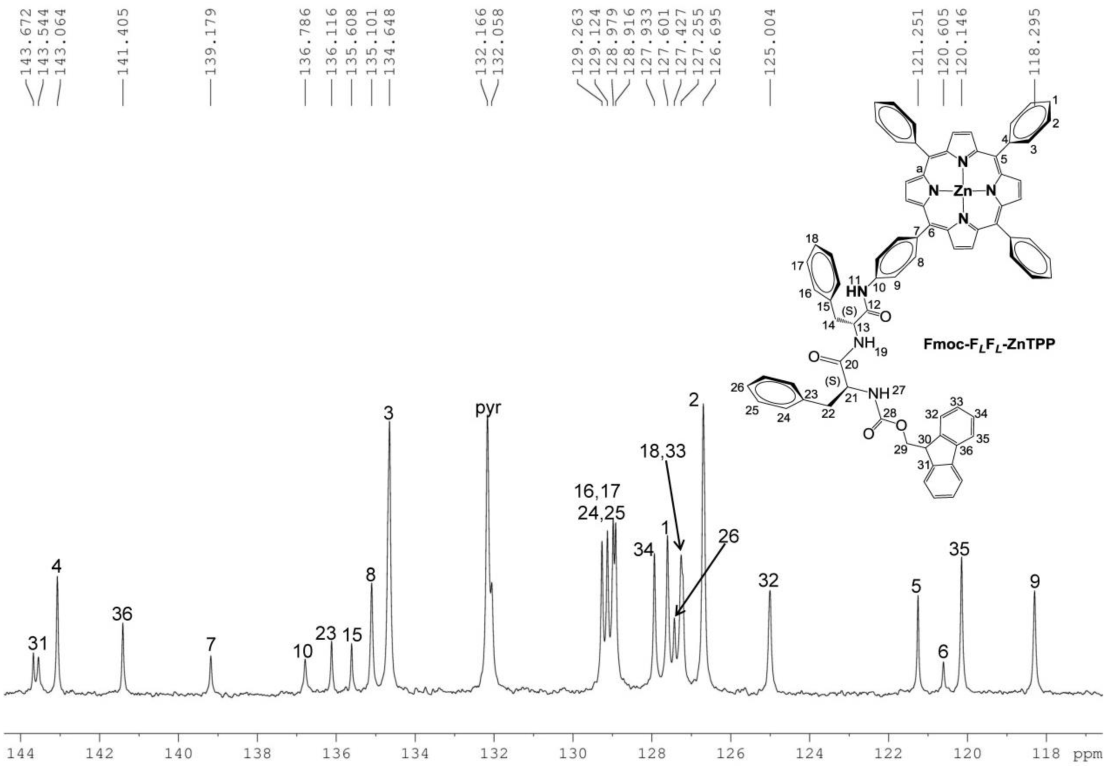

Supplementary Figure 15. Aromatic region of the ^{13}C NMR spectrum for compound **Fmoc-F$_L$F$_L$-ZnTPP** in $CDCl_3$.

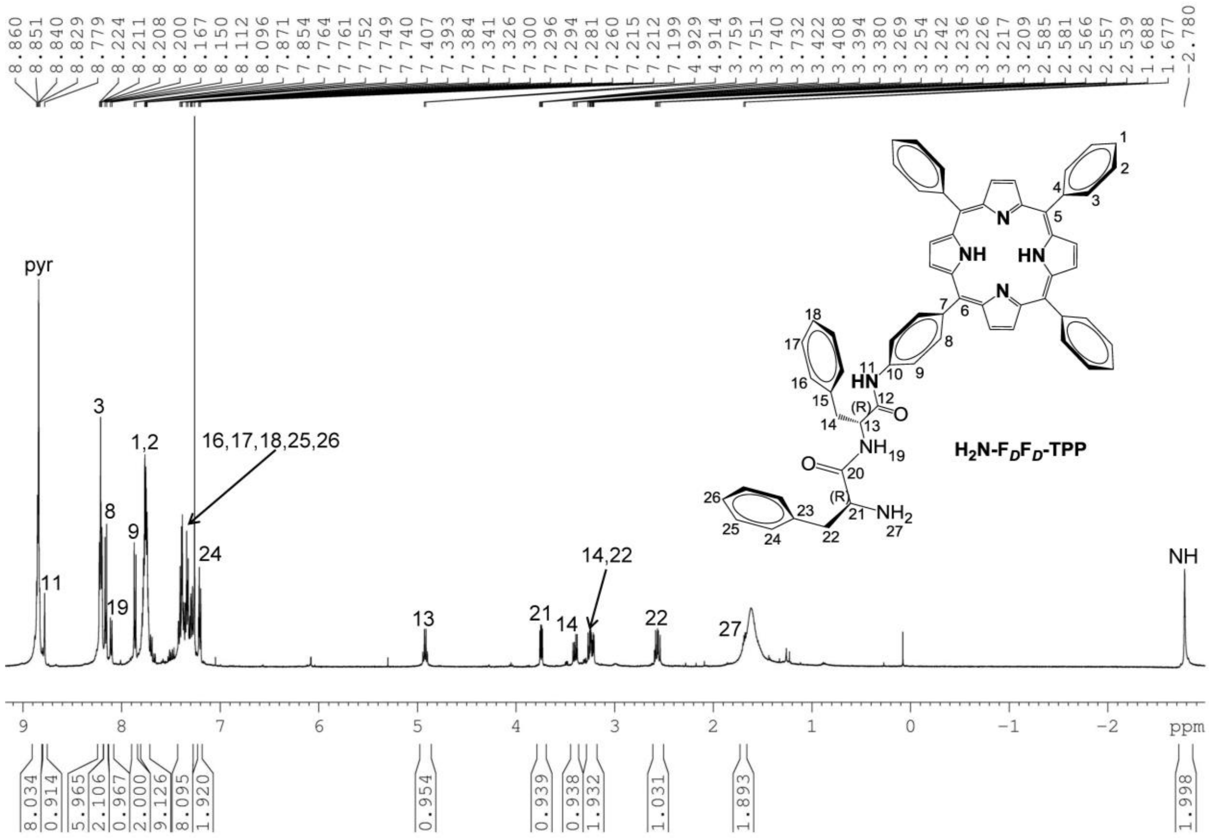

Supplementary Figure 16. ^{1}H NMR spectrum of compound **H$_2$N-F$_D$F$_D$-TPP** in $CDCl_3$.

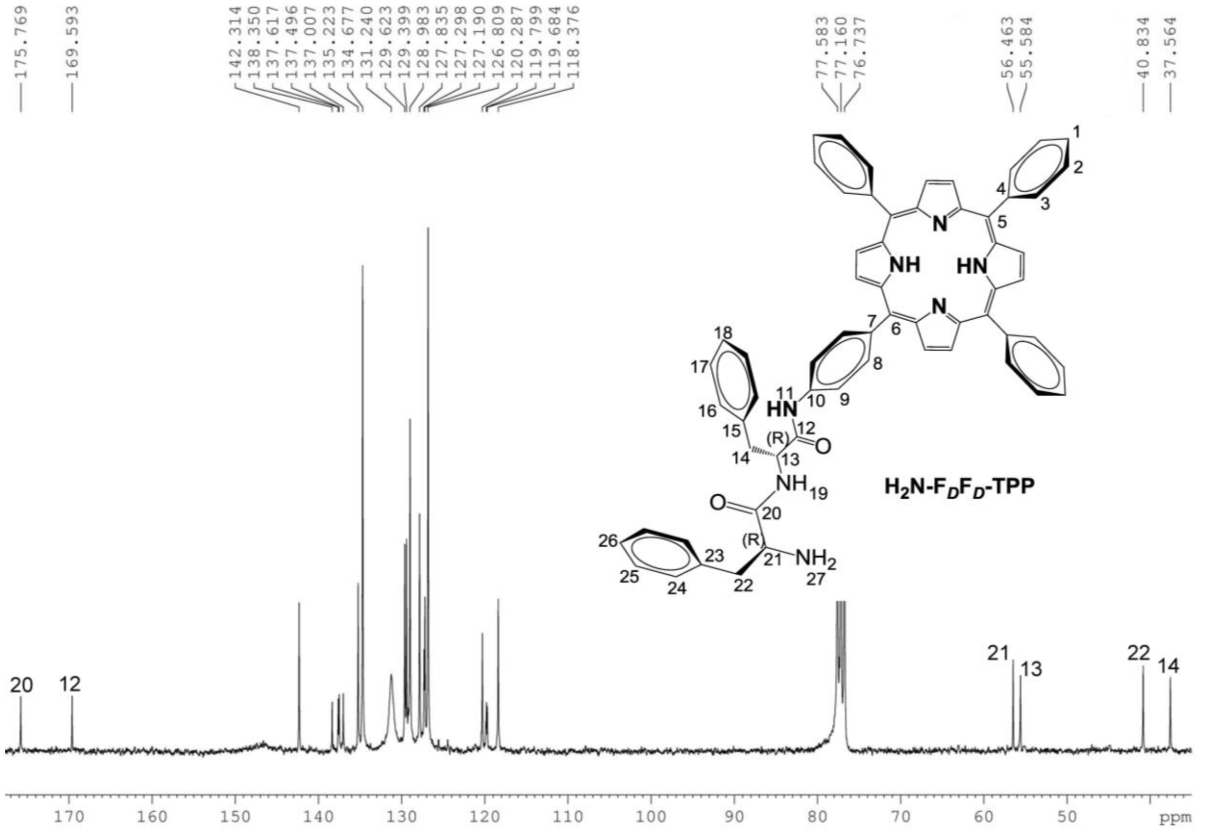

Supplementary Figure 17. ^{13}C-NMR spectrum of compound **H$_2$N-F$_D$F$_D$-TPP** in $CDCl_3$.

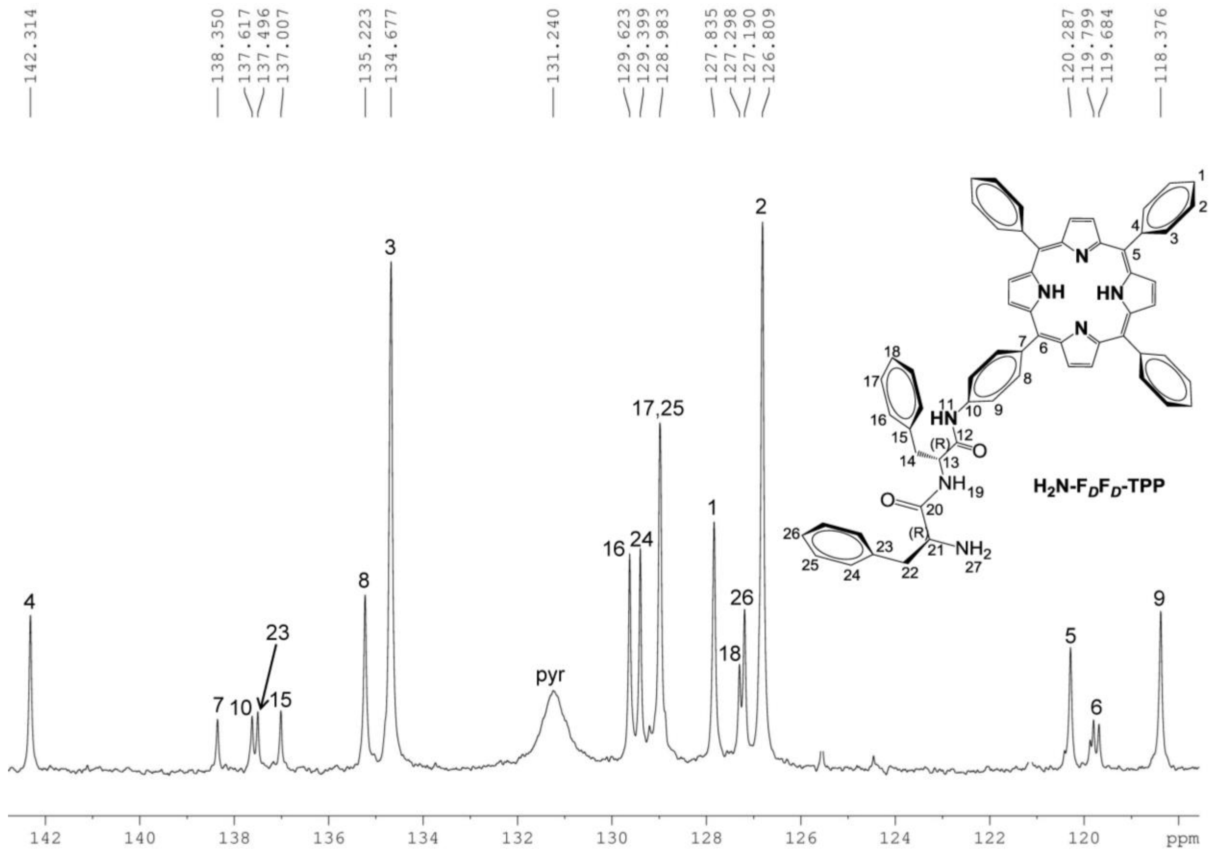

Supplementary Figure 18. Aromatic region of the ^{13}C NMR spectrum for compound **H_2N-F*D*F*D*-TPP** in $CDCl_3$.

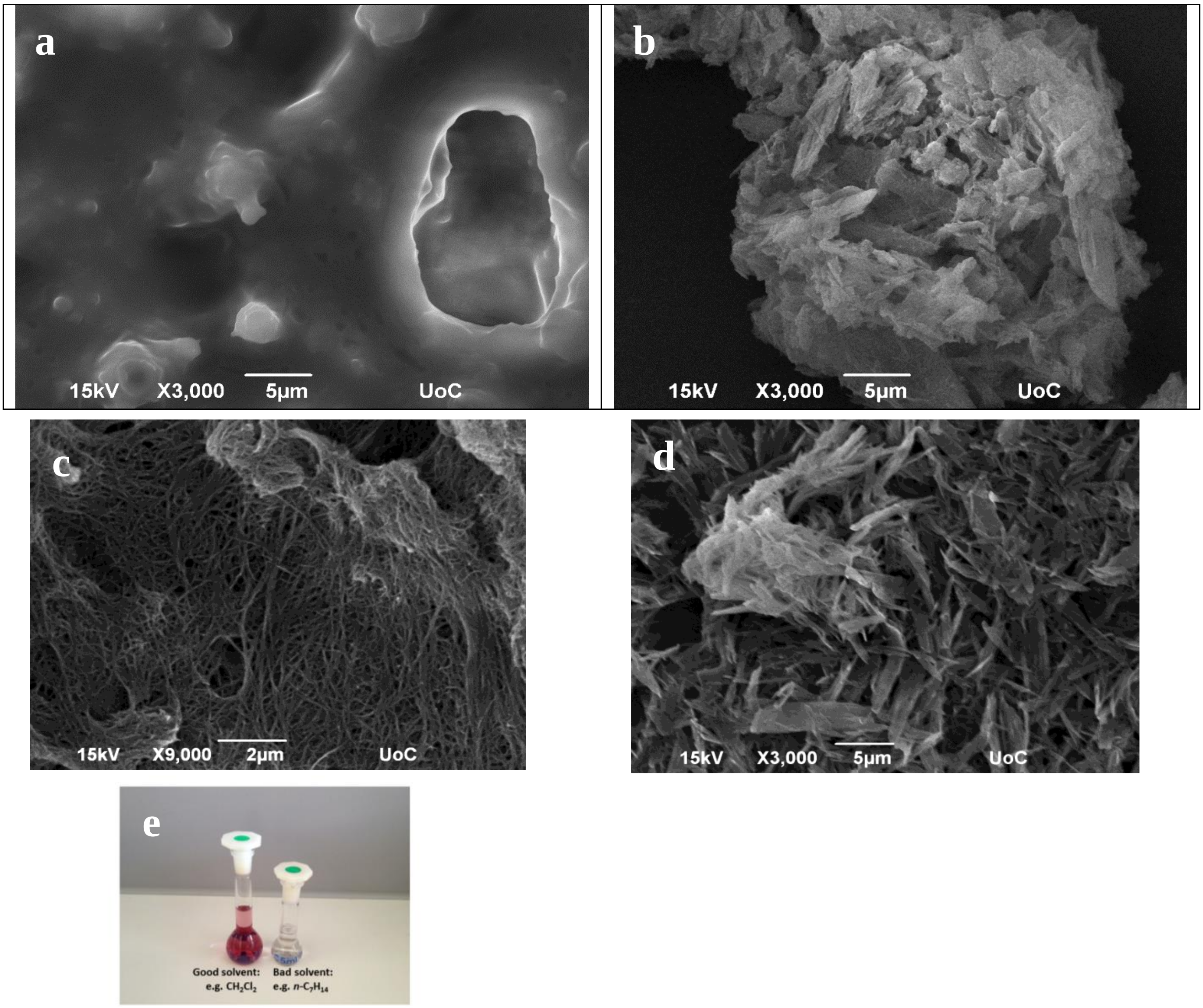

Supplementary Figure 19. Scanning Electron Microscopy (SEM) micrographs of **Fmoc-F$_{L}$F$_{L}$-TPP** in pure solvents and in CH_2Cl_2 / *n*-heptane solvent mixtures.
a: **Fmoc-F$_{L}$F$_{L}$-TPP**, 1 mM final concentration in 100% dichloromethane
b: **Fmoc-F$_{L}$F$_{L}$-TPP**, 1 mM final concentration in 100% *n*-heptane
c: **Fmoc-F$_{L}$F$_{L}$-TPP**, 1 mM final concentration in 30% CH_2Cl_2 / 70% *n*-heptane
d: **Fmoc-F$_{L}$F$_{L}$-TPP**, 1 mM final concentration in 20% CH_2Cl_2 / 80% *n*-heptane.

e:The solubility of **Fmoc-F$_{L}$F$_{L}$-TPP** in dry dichloromethane is superior to 1.2 mg in 10 mL after short sonication at room temperature. It is insoluble in dry *n*-heptane. This difference between a "good" and a "bad" solvent is useful for the injection method to induce self-assembly.

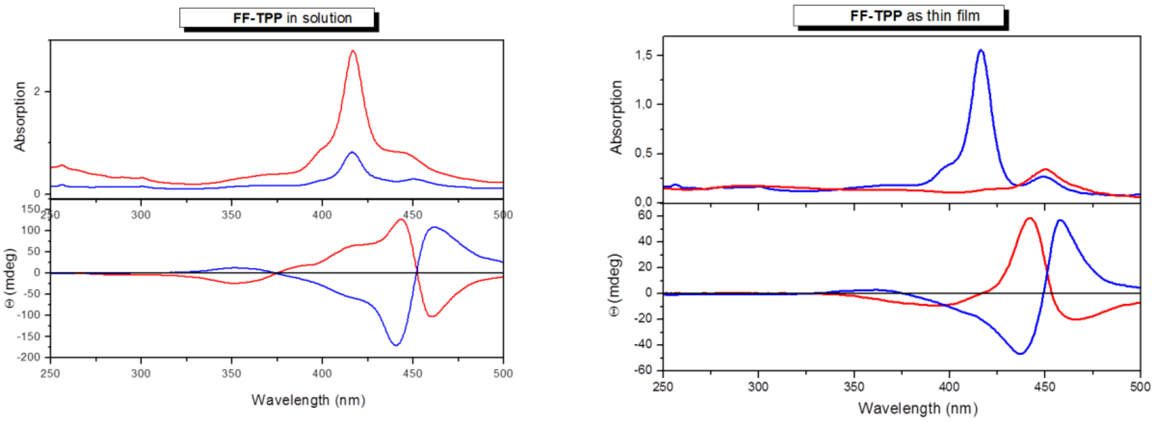

Supplementary Figure 20. Absorption and Electronic Circular Dichroism spectra of unprotected **F_LF_L-TPP** which formed a film on the cuvette walls. At left the solution spectra recorded with a path length of 1 mm. At right spectra obtained from the empty cuvette after the solution was decanted leaving a thin film visible to the eye. Note that the film from the **F_LF_L-TPP** contained a much larger amount of monomeric porphyrins with an absorption maximum at 420 nm which do not have practically an ECD contribution. The self-assemblies have an absorption maximum at 450 nm.

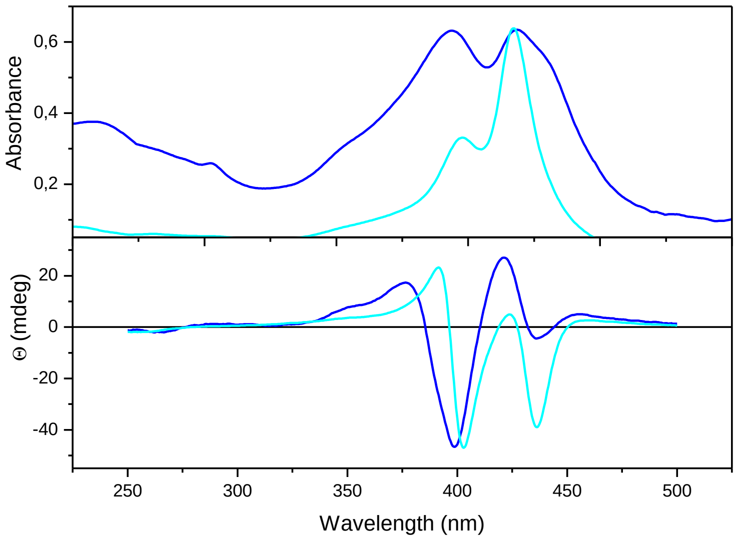

Supplementary Figure 21. Absorption and Electronic Circular Dichroism Spectra of **Fmoc-F_LF_L-TPP** (blue traces) in comparison to vertically scaled at the strongest negative Cotton effect of **Boc-F_LF_L-TPP** (cyan traces).

Stationary Fluorescence Spectra

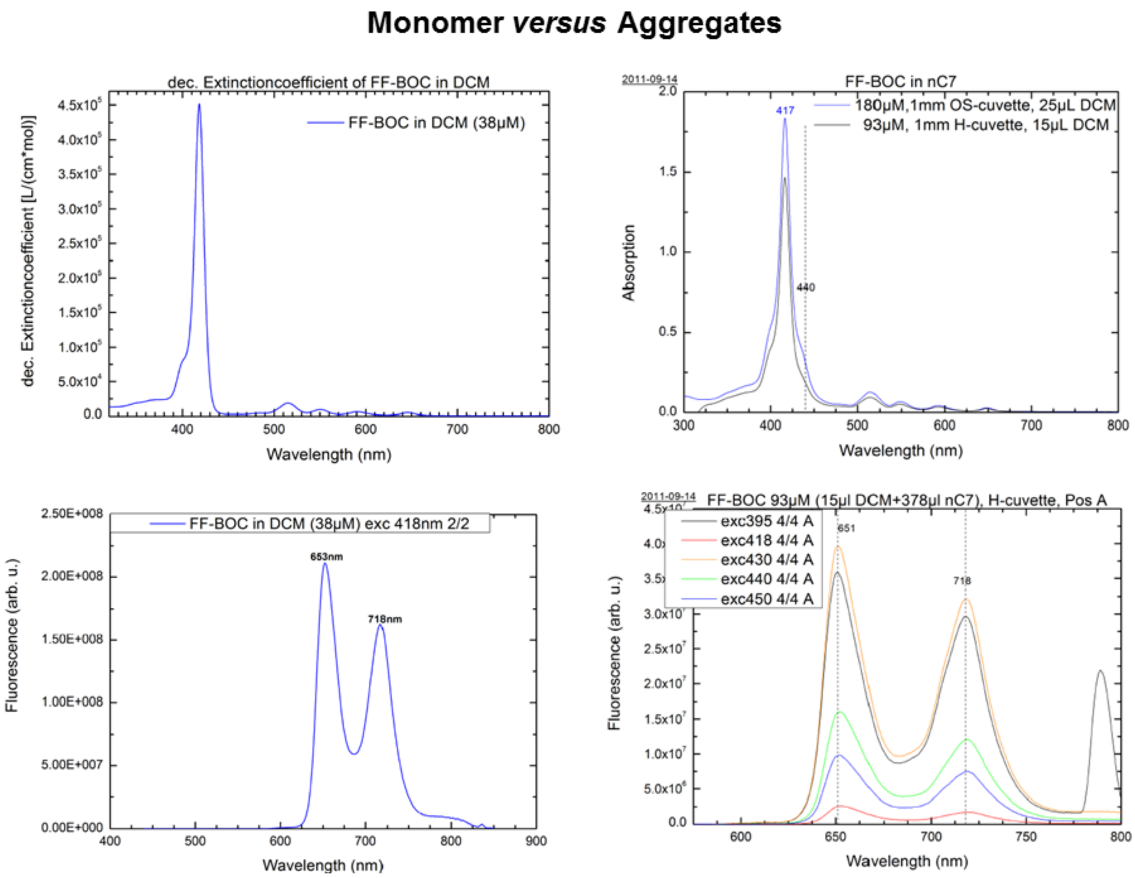

Supplementary Figure 22. Absorption spectra and stationary fluorescence spectra from **Boc-F*L*F*L*-TPP** in dry dichloromethane (at left) and by dilution in n-heptane (at right). The aggregate maxima appear as blue and red-shifted shoulders and are much smaller than for the monomer with the Soret band peaking at 417 nm. Below are fluorescence spectra with a major contribution from the monomers. At right, different excitation wavelengths were used. The maximum at 790 nm is the double of the excitation wavelength and does not originate from the sample.

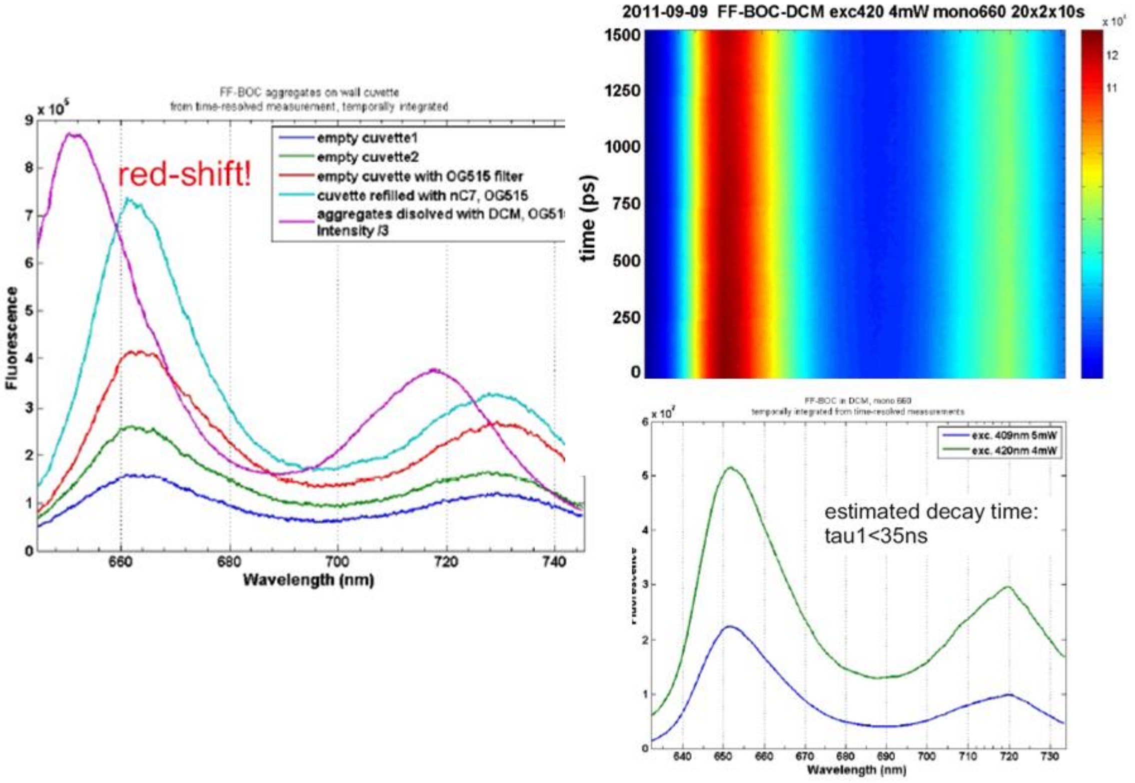

Supplementary Figure 23. At left stationary fluorescence spectra of **Boc-$F_{L}F_{L}$-TPP** obtained from the thin film remaining on the cuvette walls. These show a red shift in comparison to the monomeric spectra obtained by dissolving the film in dichloromethane (magenta trace). At right, time resolved fluorescence spectra. The X-axis is on the same scale as in the spectra obtained below for two different excitation wavelengths.

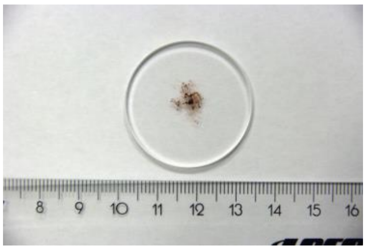

Supplementary Figure 24. Picture of the slide used on the FT-IR microscope with a ruler having the figures in cm.

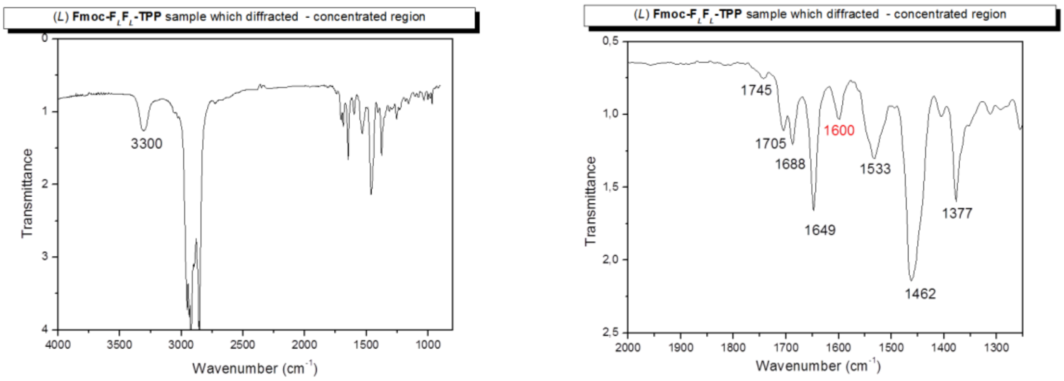

Supplementary Figure 25. FT-IR Spectra gained on the light microscope from the same **Fmoc-F*L*F*L*-TPP** sample which diffracted X-rays and from which the powder diffraction pattern was obtained. The shown spectra are from the more concentrated regions of the sample. The more diluted regions show a less pronounced 1649 band. At left is the whole spectrum showing the porphyrin N-H vibrations at ~3300 cm^{-1}. At right is an enlargement of the same spectrum with the porphyrin breathing band at 1600 cm^{-1} shown in red. The 1649 band must be due to a hydrogen bonded amidic carbonyl group as its intensity is concentration dependent.

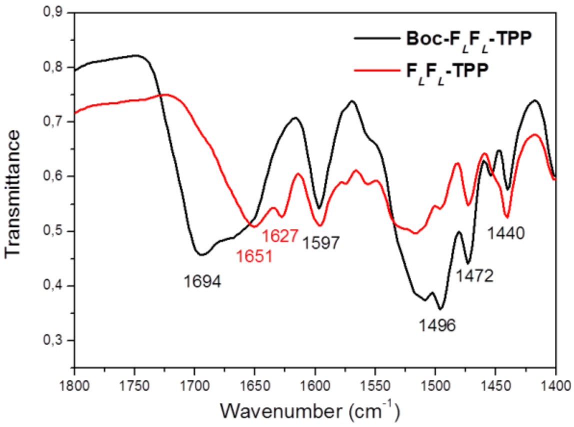

Supplementary Figure 26. FT-IR Spectra measured on a Bruker Vertex 70 instrument as KBr pellets. Black trace is from the Boc protected **Boc-F*L*F*L*-TPP** sample while the red trace is from the unprotected **F*L*F*L*-TPP**. Note the disappearance of the broad ester carbonyl bands at ca. 1694 cm^{-1}. For a similar plot comparing **Fmoc-F*L*F*L*-TPP** to **F*L*F*L*-TPP** see the main text Figures 4a and Bb.

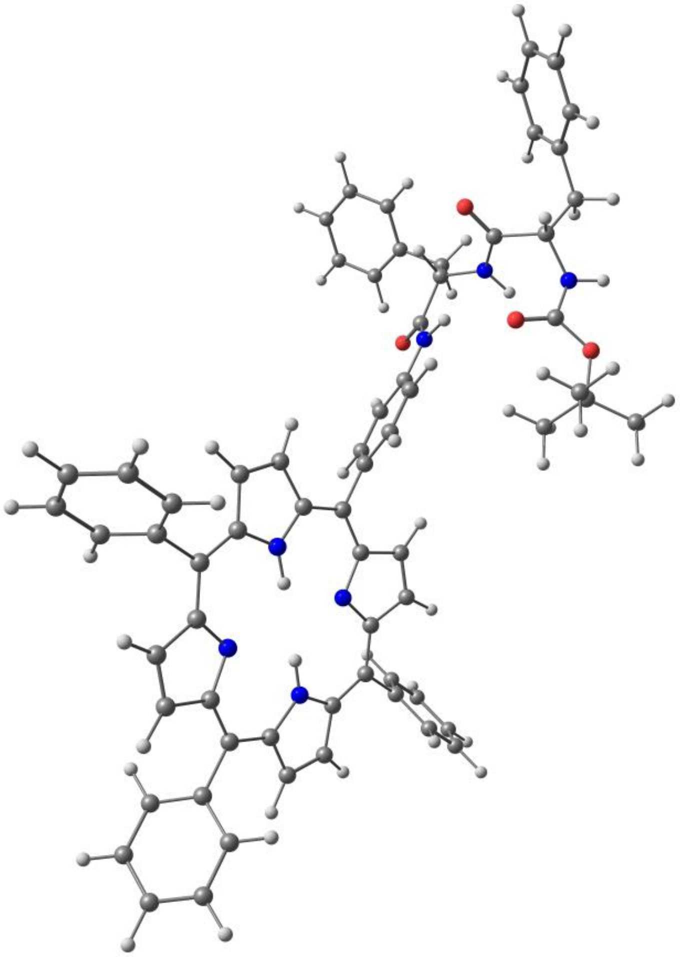

Supplementary Figure 27. Geometry of the optimized structure for the **Boc-F$_L$F$_L$-TPP** monomer.

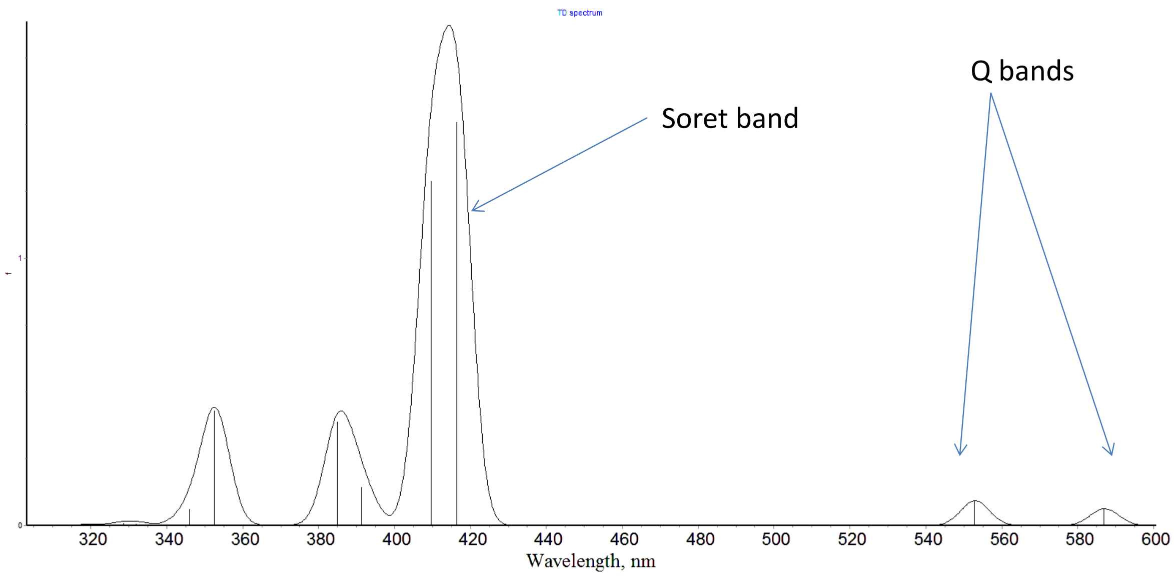

Supplementary Figure 28. TD-DFT simulated spectra of the **Boc-F$_L$F$_L$-TPP** monomer.

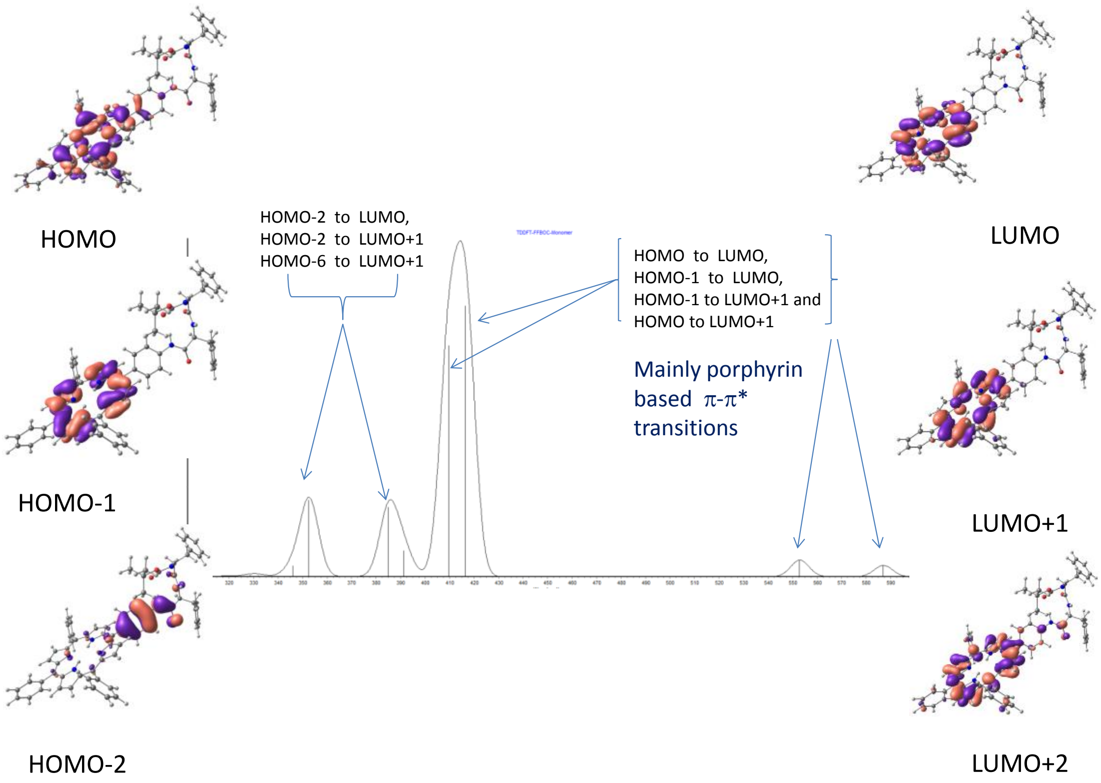

Supplementary Figure 29. Frontier Molecular Orbitals pictures and the transitions involved in absorption spectra.

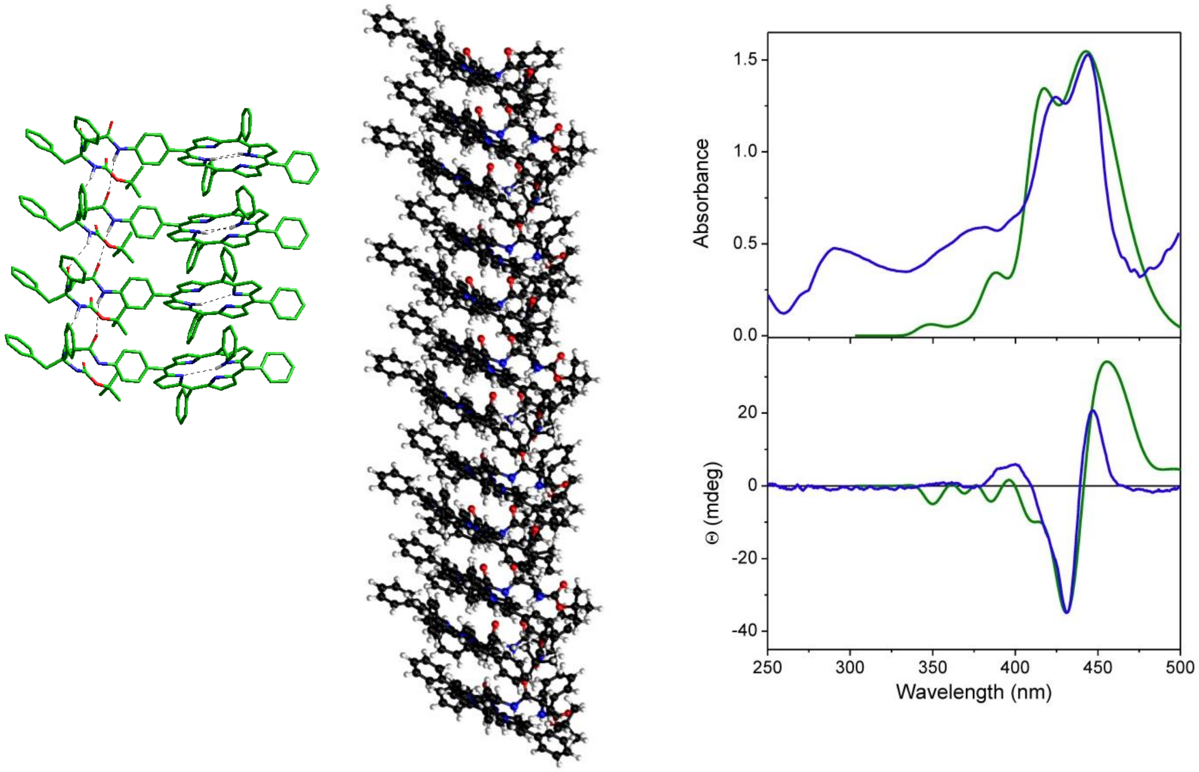

Supplementary Figure 30. Theoretical calculations on Boc-F_LF_L-TPP oligomers in vacuum that match the absorption and ECD spectra of the thin films deposited on the cuvette walls. At left: a possible tetrameric assembly of **Boc-F_LF_L-TPP**. Putative hydrogen bonds, as put into evidence by the FT-IR spectra are shown by dotted lines. Middle: a dodecamer obtained after molecular dynamics simulations. At right: calculated (green traces) absorption (top) and ECD spectra (bottom) and of the dodecamer which reproduce well the experimental spectra (blue traces) of the thin film, especially in the region of the Soret band. The ECD spectrum was scaled at the height of the negative Cotton effect and a correction of + 73 nm was applied to the calculated spectrum.

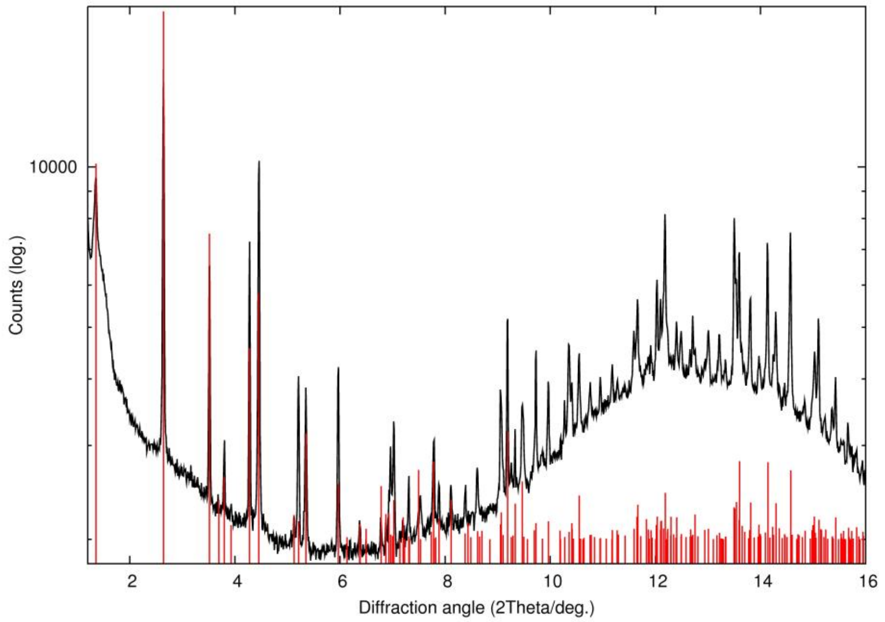

Supplementary Figure 31. Powder Diffraction Data (logarithmic scale of intensities) and the calculated reflexes as red lines for **Fmoc-F*L*F*L*-TPP**. The same sample was used as for the FT-IR studies. See also Figure 5c in the main text for a linear scaling.

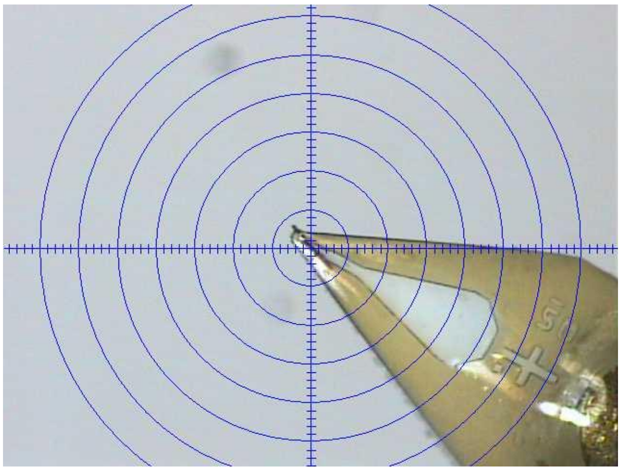

Supplementary Figure 32. A single crystal of **Fmoc-F*L*F*L*-TPP** with approximate dimensions of 12×15 ×100 µm showing the typically small size of such crystals but which diffracted well upon using synchrotron X-rays. From the same batch the powder diffraction data and FT-IR spectra were obtained.

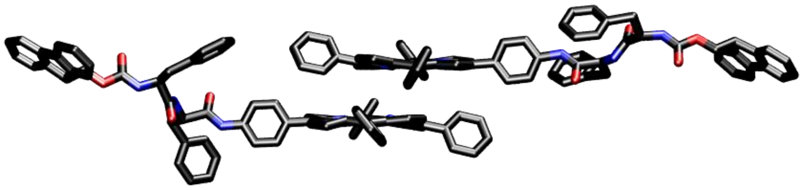

Supplementary Figure 33. A dimer in the crystal lattice of **Fmoc-F_LF_L-TPP** with close π-stacking of the porphyrin macrocycles accounting for the strong excitonic interaction.

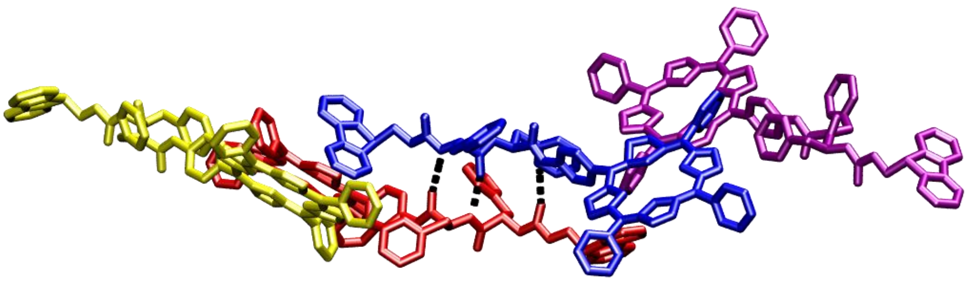

Supplementary Figure 34. Four differently coloured molecules in the crystal lattice of **Fmoc-F_LF_L-TPP** showing the triple hydrogen bonds (dashed black lines) between two adjacent FF dipeptide units.

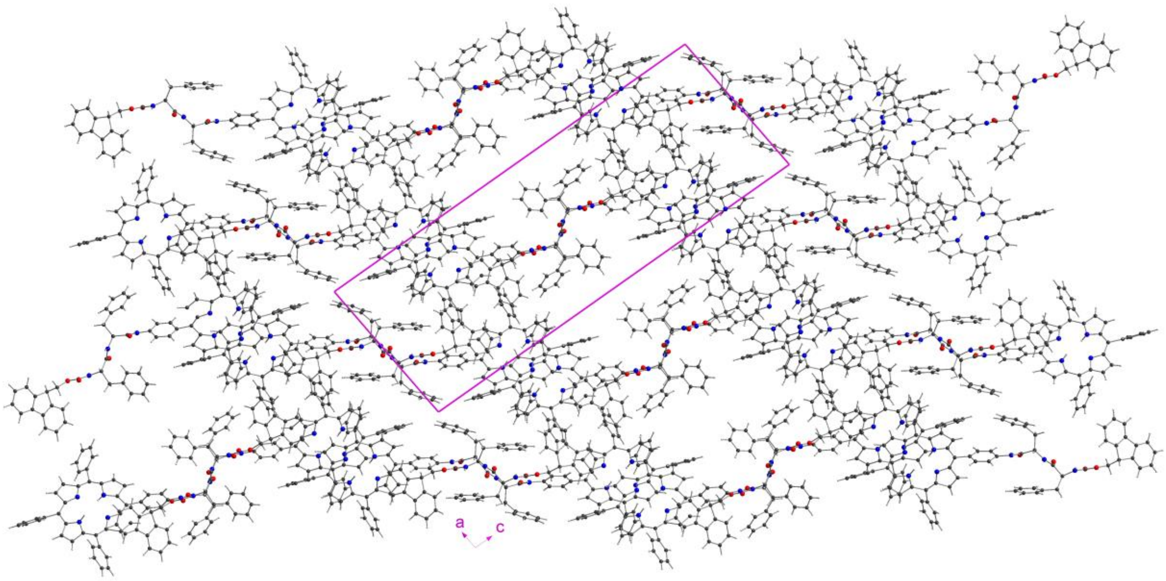

Supplementary Figure 35. A larger view of the crystal lattice viewed from the *b* axis, similarly to Figure 4f, in the main text.

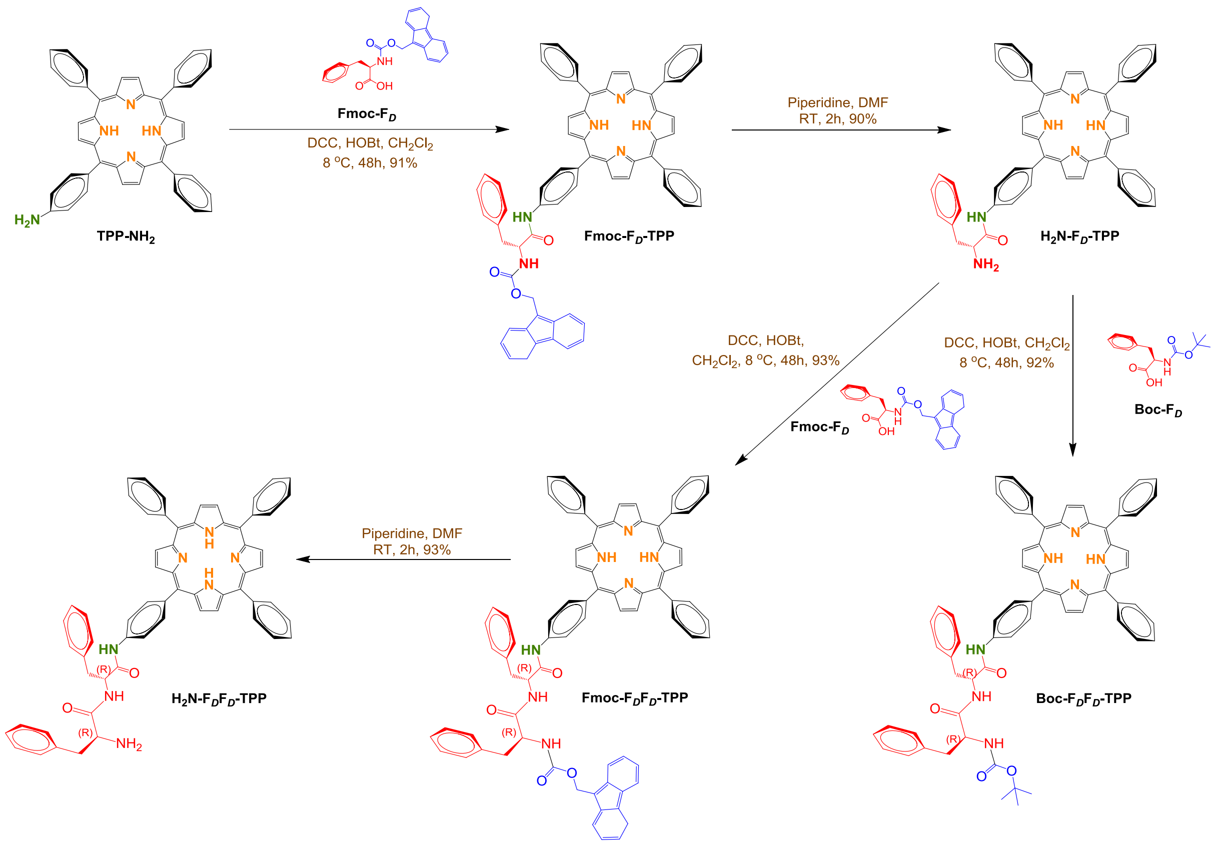

Supplementary Figure 36. Reaction conditions and yields in the synthetic procedures for the preparation of the new X-**F*D*F*D*-TPP** compounds.

Supplementary Methods

General procedure for coupling phenylalanine with the porphyrin compounds: The Boc- or Fmoc-protected phenylalanine (1 equiv) was dissolved in dry dichloromethane and the solution was cooled to 0 °C in an ice bath. *N,N'*-dicyclohexylcarbodiimide (DCC, 1.2 equiv) and 1-hydroxybenzotriazole hydrate (HOBt, 1.2 equiv) were added and the stirring was continued for 30 min at 0 °C. Then the porphyrin compound **TPP-NH$_2$** (1 equiv) was added and the resulting mixture was stirred at 8 °C for 48 h. After addition of CH_2Cl_2 the solution was washed once with water and the organic layer was dried over Na_2SO_4, filtered, concentrated, and the residue was purified by chromatography on a silica gel column.

Fmoc-F*$_D$*-TPP: Following the general procedure Fmoc-phenylalanine (**Fmoc-F*$_D$***, 50 mg, 0.13 mmol) was coupled with monoaminoporphyrin **TPP-NH$_2$** (80 mg, 0.13 mmol) by using DCC (31 mg, 0.15 mmol) and HOBt (20 mg, 0.15 mmol) in CH_2Cl_2 (6 mL). The title compound was isolated by column chromatography (silica gel, CH_2Cl_2/EtOH, 100:3 *v/v*) as a purple solid (115 mg, 91%). ^{1}H NMR ($CDCl_3$, 500 MHz): δ 9.83 (s, 1H), 8.88 (m, 8H), 8.22 (m, 8H), 8.02 (d, *J* = 8.0 Hz, 2H), 7.77 (m, 11H), 7.71 (d, *J* = 7.5 Hz, 2H), 7.36 (m, 9H), 4.06 (t, *J* = 7.8 Hz, 1H), 3.91 (m, 1H), 3.52 (m, 2H), 2.95 (m, 1H), 2.61 (d, *J* = 7.8 Hz, 2H), -2.74 (s, 2H); ^{13}C NMR ($CDCl_3$, 75 MHz): δ 172.9, 146.8, 142.3, 141.1, 138.1, 137.9, 137.6, 135.3, 134.7, 131.2, 129.5, 129.1, 127.9, 127.2, 127.1, 126.8, 125.6, 120.3, 119.8, 119.7, 117.9, 63.2, 57.2, 45.0, 41.0. HR-MS (MALDI-TOF): m/z calculated for $C_{68}H_{51}N_6O_3$: 999.4023 $[M+H]^+$; found: 999.4031. Elemental analysis calculated for $C_{68}H_{50}N_6O_3$: C, 81.74; H, 5.04; N, 8.41. Found: C, 81.68; H, 5.12; N, 8.38.

H$_2$N-F*$_D$*-TPP: Piperidine (1.4 mL) was added to a solution of **Fmoc-F*$_D$*-TPP** (120 mg, 0.12 mmol) in DMF (7 mL) and the mixture was stirred at room temperature for 2 h. The solvent was then removed under vacuum and the solid was dissolved in CH_2Cl_2 (100 mL), washed with water (2 x 100 mL), dried over Na_2SO_4, filtered, and concentrated under vacuum. The title compound was isolated by column chromatography (silica gel, CH_2Cl_2/EtOH, 100:1.5 *v/v*) as a purple solid (84 mg, 93%). ^{1}H NMR ($CDCl_3$, 500 MHz): δ 9.84 (s, 1H), 8.90 (d, *J* = 4.5 Hz, 2H), 8.86 (m, 6H), 8.22 (m, 8H), 8.02 (d, *J* = 8.0 Hz, 2H), 7.77 (m, 9H), 7.39 (m, 5H), 3.91 (dd, J_1 = 10.0 Hz, J_2 = 4.0 Hz, 1H), 3.54 (dd, J_1 = 13.5 Hz, J_2 = 4.0 Hz, 1H), 2.95 (dd, J_1 = 13.5 Hz, J_2 = 10.0 Hz, 1H), 1.84 (m, 2H), -2.75 (s, 2H); ^{13}C NMR ($CDCl_3$, 75 MHz): 172.9, 142.3, 138.1, 137.9, 137.6, 135.3, 134.7, 131.2, 129.5, 129.1, 127.9, 127.2, 127.1, 126.8, 120.3, 119.8, 117.9, 57.2, 41.0. HR-MS (MALDI-TOF): m/z calculated for $C_{53}H_{41}N_6O$: 777.3342 $[M+H]^+$; found: 777.3337. Elemental analysis calculated for $C_{53}H_{40}N_6O$: C, 81.93; H, 5.19; N, 10.82. Found: C, 81.88; H, 5.23; N, 10.79.

Boc-F*$_D$*F*$_D$*-TPP: The general procedure was followed for coupling Boc-phenylalanine **Boc-F*$_D$*** (38 mg, 0.13 mmol) with **H$_2$N-F*$_D$*-TPP** (100 mg, 0.13 mmol) by using DCC (30 mg, 0.15 mmol) and HOBt (20 mg, 0.15 mmol) in CH_2Cl_2 (6 mL). The title compound was isolated by column chromatography (silica gel, CH_2Cl_2/EtOH, 100:4 *v/v*) as a purple solid (122 mg, 92%). ^{1}H NMR ($CDCl_3$, 500 MHz): δ 8.87 (m, 8H), 8.61 (br s, 1H), 8.22 (d, *J* = 6.2 Hz, 6H), 8.16 (d, *J* = 8.3 Hz, 2H), 7.99 (m, 2H), 7.76 (m, 9H), 7.34 (m, 8H), 7.17 (br s, 2H), 6.51 (br s, 1H), 5.02 (m, 1H), 4.90 (br s, 1H), 4.41 (m, 1H), 3.52 (br s, 1H), 3.13 (m, 3H), 1.37 (s, 9H), -2.75 (s, 2H); ^{13}C NMR ($CDCl_3$, 75 MHz): δ 171.4, 169.2, 142.3, 138.3, 137.7, 136.3, 135.9, 135.1, 134.7, 131.2, 129.5, 129.4, 129.2, 129.1, 127.8, 127.7, 127.5, 126.8, 120.3, 119.9, 118.5, 81.4, 56.7, 54.3, 37.6, 37.3, 28.3. HR-MS (MALDI-TOF): m/z calculated for $C_{67}H_{58}N_7O_4$: 1024.4550 $[M+H]^+$; found: 1024.4544. Elemental analysis calculated for $C_{67}H_{57}N_7O_4$: C, 78.57; H, 5.61; N, 9.57. Found: C, 78.55; H, 5.68; N, 9.61.

Fmoc-F_DF_D-TPP: Following the general procedure **Fmoc-F_D** (25 mg, 0.06 mmol) was coupled with **H_2N-F_D-TPP** (45 mg, 0.06 mmol) by using DCC (14 mg, 0.07 mmol) and HOBt (9 mg, 0.07 mmol) in CH_2Cl_2 (3 mL). The title compound was isolated by column chromatography (silica gel, CH_2Cl_2/EtOH, 100:4 *v/v*) as a purple solid (68 mg, 93%). ^{1}H NMR ($CDCl_3$, 500 MHz): δ 8.84 (m, 8H), 8.41 (s, 1H), 8.22 (m, 6H), 8.15 (m, 2H), 7.89 (m, 2H), 7.76 (m, 11H), 7.51 (t, J = 6.5 Hz, 2H), 7.34 (m, 10H), 7.23 (m, 4H), 6.59 (d, J = 6.5 Hz, 1H), 5.20 (d, J = 4.4 Hz, 1H), 4.95 (m, 1H), 4.47 (m, 2H), 4.39 (m, 1H), 4.17 (t, J = 6.8 Hz, 1H), 3.30 (d, J = 6.5 Hz, 2H), 3.20 (m, 1H), 3.00 (m, 1H), -2.77 (s, 2H); ^{13}C NMR ($CDCl_3$, 75 MHz): δ 171.3, 169.0, 156.7, 143.6, 142.3, 141.5, 138.4, 136.5, 135.8, 135.2, 134.7, 131.3, 129.4, 129.3, 129.2, 129.1, 128.0, 127.8, 127.7, 127.5, 127.3, 126.8, 125.1, 125.0, 120.3, 120.2, 119.7, 118.4, 67.5, 56.8, 55.1, 47.2, 37.9, 37.6. HR-MS (MALDI-TOF): m/z calculated for $C_{77}H_{60}N_7O_4$: 1146.4707 $[M+H]^+$; found: 1146.4718. Elemental analysis calculated for $C_{77}H_{59}N_7O_4$: C, 80.68; H, 5.19; N, 8.55. Found: C, 80.72; H, 5.15; N, 8.49.

H_2N-F_DF_D-TPP: Porphyrin **Fmoc-F_DF_D-TPP** (33 mg, 0.03 mmol) was dissolved in DMF (2 mL), piperidine (0.4 mL) was added and the solution stirred at room temperature for 2 h. The solvent was then removed under vacuum and the solid was dissolved in CH_2Cl_2 (30 mL), washed with water (2 x 30 mL), dried over Na_2SO_4, filtered, and concentrated under vacuum. The title compound was isolated by column chromatography (silica gel, CH_2Cl_2/EtOH, 100:4 *v/v*) as a purple solid (25 mg, 93%). ^{1}H NMR ($CDCl_3$, 500 MHz): δ 8.84 (m, 8H), 8.78 (s, 1H), 8.21 (m, 6H), 8.16 (d, J = 8.4 Hz, 2H), 8.10 (d, J = 7.9 Hz, 1H), 7.86 (d, J = 8.4 Hz, 2H), 7.75 (m, 9H), 7.34 (m, 8H), 7.21 (m, 2H), 4.92 (q, J = 7.6 Hz, 1H), 3.75 (dd, J_1 = 9.5 Hz, J_2 = 4.0 Hz, 1H), 3.40 (dd, J_1 = 14.0 Hz, J_2 = 7.0 Hz, 1H), 3.24 (m, 2H), 2.57, (m, 1H), 1.68 (m, 2H), -2.78 (s, 2H); ^{13}C NMR ($CDCl_3$, 75 MHz): δ 175.8, 169.6, 142.3, 138.4, 137.6, 137.5, 137.0, 135.2, 134.7, 131.2, 129.6, 129.4, 129.0, 127.8, 127.3, 127.2, 126.8, 120.3, 119.8, 119.7, 118.4, 56.5, 55.6, 40.8, 37.6. HR-MS (MALDI-TOF): m/z calculated for $C_{62}H_{50}N_7O_2$: 924.4026 $[M+H]^+$; found: 924.4031. Elemental analysis calculated for $C_{62}H_{49}N_7O_2$: C, 80.58; H, 5.34; N, 10.61. Found: C, 80.52; H, 5.36; N, 10.66.

Zinc metalation of Fmoc- F_LF_L-TPP free base

Fmoc-F_LF_L-ZnTPP: A mixture of porphyrin **Fmoc-F_LF_L-TPP** (20 mg, 0.017 mmol) and zinc acetate dihydrate (50 mg, 0.23 mmol) in CH_2Cl_2 (7 mL) and MeOH (2 mL) was stirred at room temperature overnight. The solvent was removed in a rotary evaporator and the desired compound was isolated by column chromatography (silica gel, CH_2Cl_2/EtOH, 100:3 v/v) as a purple solid (19 mg, 90%). ^{1}H NMR ($CDCl_3$, 500 MHz): δ 8.94 (m, 8H), 8.22 (d, J = 6.1 Hz, 6H), 8.09 (d, J = 8.1 Hz, 2H), 7.73 (m, 12H), 7.58 (m, 2H), 7.41 (m, 2H), 7.33 (m, 2H), 7.19 (m, 8H), 6.72 (d, J = 6.9 Hz, 2H), 6.82 (br s, 2H), 6.12 (br s, 1H), 4.50 (br s, 1H), 4.22 (m, 2H), 4.05 (t, J = 6.6 Hz, 1H), 3.86 (br s, 1H), 3.76 (br s, 1H), 2.81 (d, J = 6.4 Hz, 2H), 2.39 (br s, 2H); ^{13}C NMR ($CDCl_3$, 75 MHz): δ 170.5, 168.3, 156.1, 150.3, 143.7, 143.5, 143.1, 141.4, 139.2, 136.8, 136.1, 135.6, 135.1, 134.6, 132.2, 132.1, 129.3, 129.1, 129.0, 128.9, 127.9, 127.6, 127.4, 127.3, 126.7, 125.0, 121.3, 120.6, 120.1, 118.3, 67.3, 56.0, 54.7, 47.2, 37.3. HR-MS (MALDI-TOF): m/z calculated for $C_{77}H_{58}N_7O_4Zn$: 1208.3845 $[M+H]^+$; found: 1208.3841. Elemental analysis calculated for $C_{77}H_{57}N_7O_4Zn$: C, 76.45; H, 4.75; N, 8.11. Found: C, 76.51; H, 4.69; N, 8.19.

Theoretical calculations on the Boc-F_LF_L-TPP monomer in vacuum and CH_2Cl_2

In order to obtain a better insight into the electronic properties of the **Boc-F_LF_L-TPP** monomer we performed calculations using 6-31G(d) basis sets. The geometry optimized structure possesses a nearly planar porphyrin ring (Figure S24). TD-DFT analysis (using dichloromethane as solvent phase) of the **Boc-F_LF_L-TPP** monomer porphyrin reveals the presence of an intense Soret band in the region of 410 nm to 417 nm along with two Q bands at 553nm and 587 nm corresponds to porphyrin based π-π* transitions (Figure S25). The absorptions at 391 nm, 385 nm are due to the transitions from amino acid fragments to the porphyrin moiety as observed from the molecular orbital picture (Figure S26). The HOMO and LUMO fragments are located mainly on the porphyrin moiety (Figure S26).

Powder diffraction data and comparison with the single crystal X-ray data

Based on the positions of the first 10 reflections the lattice constants were evaluated. Compared to the CIF-file from the single crystal the powder cell refinement gave slightly different cell lengths and a slightly lower beta-angle (see below):

Celref: FFFMOC-Cyc6-sum0506.2th

Number of reflections : 10
Refinement constraints : NO

Final values : (Standard errors on 2nd line)

Zero	Lambda	a	b	c	alpha	beta	gamma
**-.00031**	**.99917**	**16.6378**	**9.9500**	**44.6149**	**90.00**	**92.50**	**90.00**
.00051	.00000	.0048	.0024	.0000	.00	.00	.00

Volume (A**3) : 7378.817

Number of cycles: 3

H	K	L	2Th(obs)	2Th-Zero	2Th(Calc)	diff.
0	0	1	1.2815	1.2818	1.2844	-.0026
0	0	2	2.5715	2.5718	2.5690	.0028
1	0	0	3.4455	3.4458	3.4446	.0012
1	0	1	3.7285	3.7288	3.7285	.0003
-1	0	2	4.2075	4.2078	4.2069	.0009
1	0	2	4.3855	4.3858	4.3864	-.0006
-1	0	3	5.0545	5.0548	5.0566	-.0018
0	0	4	5.1395	5.1398	5.1393	.0006
1	0	3	5.2795	5.2798	5.2806	-.0008
0	1	1	5.8975	5.8978	5.8978	.0000

Sqrt(Sum(Th O-C)**2)/(Nref-Npar)) = .000929
Sqrt(Sum(2Th O-C)**2)/(Nref-Npar)) = .000465

The simulated powder data was calculated using *Crystallographica*[1] and the CIF. (but with the cell parameters based on the above). A comparison of the powder pattern generated by *Crystallographica* and that generated by Mercury shows high consistency.

For the crystallization trials in the 10 cm tubes, three limpid layers could be discerned: a lower dichloromethane very dark purple coloured layer up to ~8-10 mm, an intermediate layer of a 1:1 (v:v) dichloromethane and cyclohexane up to ~16-20 mm and a third colourless cyclohexane layer filling the tube. If turbidity appeared rapidly within the second or third layer, this usually led to amorphous material which did not diffract X-rays. On the other hand if the three layers remained limpid, slow diffusion over a period of several months usually yielded in microcrystals.

$C_{89}H_{83}N_7O_4$, 1314.62 g mol^{-1}, monoclinic, $P2_1$, a = 16.43(3), b = 10.012(15), c = 44.30(9) Å, β = 95.65(8)°, Z = 4, V = 7252(23) Å^3, T = 100(2) K, ρ_{calc} = 1.204 g cm^{-3}, $F(000)$ = 2792, μ = 0.065 mm^{-1}; 10318 data, of which 8544 unique (R_{int} = 0.0885), 495 parameters, 204 restraints, final wR_2 = 0.6053, S = 1.485 (all data), R_1 (2524 data with I > 2σ(I)) = 0.2501.

Supplementary Reference

---

[1] *Crystallographica* – a software toolkit for crystallography, *J. Appl. Cryst*. **30**, 418-419 (1997).
